# Measuring the effects of differentially intense information on political opinions

**DOI:** 10.1371/journal.pone.0333129

**Published:** 2025-11-26

**Authors:** Claudia Zucca

**Affiliations:** Tilburg University, Jheronimus Academy of Data Science, ’s-Hertogenbosch, The Netherlands; University of Glasgow, UNITED KINGDOM OF GREAT BRITAIN AND NORTHERN IRELAND

## Abstract

The field of public opinion has already extensively addressed how political opinions form and change. However, we still need to find out why some information flows affect opinions more effectively than others. The concept of intensity is employed to assess the differential penetrating power attributed to different political information flows. A measure of the intensity of information flows is presented and tested on British respondents with a survey experiment that employs vignettes with information flows concerning the former British Conservative Prime Minister Theresa May. This study finds that the measure of intensity is able to identify information flows that have a larger probability of affecting opinions. Information flows characterised by higher intensity are more likely than information flows characterised by low intensity to influence opinion formation about the former British leader. Still, respondents’ pre-existing political awareness and predispositions condition the effect of information flow intensity in agreement with established theories in public opinion. Different political awareness and predispositions are associated with different reactions to the intensity of information flows. Finally, the study finds that effects can be observed only if the information flow is intense enough, independent of the respondents’ attitudes.

## Introduction

Seminal studies in several disciplines, such as political science, public opinion, communication, and psychology, have provided considerable cross-disciplinary evidence to explain the way information is transmitted and received, in addition to explaining the processes through which it influences opinion formation [1]. This field is so prolific that it has produced about 4500 studies since 1935, which qualifies this debate as one of the most vibrant of all time [2]. In the last thirty years, scholars have made remarkable progress so that “researchers no longer ask whether communications shape opinions, but rather when and how” [3, p. 875].

Although it is intuitive to claim that not every information flow affects its audience in the same way since, for instance, two political ads do not have the same consequences on the population, more effort is required to understand why it is so. We already know that the source of information matters [4], as much as the channel used to transmit it [5] and that elites are more capable of shifting mass opinion than ordinary citizens [6]. We also know that the agenda setting function of the mass media [7] and the priming [8] and framing of news [9] massively affect their salience. Information flows can also have different purposes, for example, when we consider persuasive and cueing messages [10]. However, what confers on particular information flows a higher likelihood of being received and influencing opinions compared to others? This study addresses this question with a measure of the intensity of an information flow.

Zaller [10] employs the concept of intensity to assess the differential penetrating power of information flows produced by political elites. Intensity plays a role within the Receive Accept Sample (RAS) model, where individuals process information according to their political awareness and predispositions [10]. This study builds on Zaller’s work and provides a methodology for measuring intensity by combining thematic analysis [11] and Shannon entropy [12].

The article proceeds as follows. In the Theoretical background section, we introduce the relevant public opinion and political communication literature to place this research into context. In the section titled ‘The intensity of information flow,’ Zaller’s concept of the intensity of an information flow is introduced together with the research questions. Section ‘Measuring the intensity of the information flow’ presents a novel methodology to measure intensity. The section ‘Case study and hypotheses’ presents the case study employed to test the measure. An experimental design [13] that focuses on the formation of opinions about the former British Prime Minister Theresa May is used for the scope. The section after provides details on the methodology employed in this study, including the experimental design. Respondents were either exposed to vignettes containing information flows characterised by different intensities or assigned to a control group. The last three sections present the data used to assess the effect of the measure of intensity, the results, and their discussion.

This study finds that the intensity measure is capable of identifying information flows that have a higher probability of affecting opinions. The results suggest that political information flows characterised by high intensity have higher penetrating power than those characterised by low intensity. Political awareness and predisposition also explain the penetrating power of information, as [10] already claimed, but only if the information is intense enough.

Further research is needed to explore the validity and reliability of the intensity measure introduced in this study to a greater extent. Still, these results suggest that the intensity of the information is a robust way to predict the effects of the information on the change in opinion.

## Theoretical background

Scholars within the field of psychology agree broadly in explaining the mechanisms through which human brains receive or reject information with the use of heuristic cues and the pre-existence of attitudes [14–18]. The early work of [4,6,19] showed that changing attitudes in a political context is difficult since political opinions are formed in a family context when people are young. Pre-existing attitudes can be challenged only by authoritative sources or elites [6], and they might prevent us from actual knowledge of the social reality that could bias our decision making [20]. Since information is received through heuristic cues and we filter it with our attitudes, we cannot aspire to unbounded rationality [20].

Other scholars are more inclined to believe in attitude change. [21] explains this process with the intention of re-balancing a state of cognitive dissonance, while [22] attributes it to a personal motivation driven by accuracy or directional goals. [23,24] and [25] explain attitude change with dual-process models, stressing the difference between the cognitive and emotional constitutive parts of attitudes in opposition to the behavioural one. Alternatively, a different and more heterogeneous scholarship focused on the relevance of the message’s features for opinion formation. Agenda Setting [7,26], Priming [8,27,28], and Framing [9,29] explain the power of mass media and elites in temporarily shifting the salience of attitudes in people’s minds [28] (temporary opinion shift).

It is now widely accepted that the medium chosen to deliver a message has an impact on the final effect on the audience [5,30,31]. The effects of messages are also explained with the authoritativeness of political parties [32], or with the choice of language [33]. Theories focusing on reception and processing are connected to the literature on the message’s features through political awareness and considerations. Attitudes are activated by political information and turn into political awareness (knowledge). Considerations are the reasons that might induce people to make up their minds either way about political issues [10]. These considerations are attitudes (if the respondent has any concerning a specific topic), enlightened by new information.

Attitudes, such as interest in politics and political awareness, interfere with the reception of information and the features of the message [10] since they funnel new information and ultimately affect the formation of opinions. These variables are widely employed in studies that explain opinion formation [34,35].

In summary, the process of opinion formation under the reception of new information has been widely explored. However, there has been limited attention to the features that characterise the actual content that is received. [10] discussed this problem and used the concept of intensity to distinguish between the effects of different information, to which he refers as information flows. In the following section, we introduce the concept of the intensity of an information flow and, in the one after it, a methodology to measure it.

## The intensity of information flows

John Zaller [10] employs intensity as an attribute of information flows expressed by a political elite to explain its power to persuade individuals and influence them. When the intensity of the information flow is low, individuals are unlikely to be affected by exposure to information; conversely, when the intensity of the information flow is high, the probability of being influenced increases [, p. 14; 155-158; 267].

John Fiske [36] comes to the same conclusion with the concept of redundancy. If several messages convey the same theme repeatedly (redundantly), it is more likely that the information is received. Moreover, redundant information flows are more likely to sound familiar to the receiver, since the receiver can find more arguments that are already familiar inside the new information. Fiske calls this phenomenon predictability and connects predictable messages with penetrating power [, p. 10]. The argument can also be supported in parallel using the Elaboration Likelihood Model (ELM) [23]. The ELM theorises a central and a peripheral route to receive information. Both routes process several inputs at the same time. Thus, if information flows are intense or redundant, individuals would have higher chances of receiving it, either through the central or the peripheral route.

Zaller measures the intensity of an information flow as “count of the number of media reports and the direction where the report pushes opinions" [, p. 14]. However, by focusing on the information flows containing the media coverage of a politically relevant issue, Zaller does not capture the intensity of information but instead measures the intensity of the media coverage. Moreover, this definition allows for the analysis of the intensity of very large information flows, and we aim to consider smaller bodies of information, too.

For this reason, instead of considering the number of media reports in an information flow, we consider the number of themes and their frequencies. The following section explains the methodology employed to measure it. After introducing this definition and its measure, we experimentally test the validity of the intensity of the information measure. [10] conceive intensity as a measure of the power of persuasion attributed to an information flow. Hence, a first research question is specified:

- Q1: Do differentially intense information flows have differential effects on forming opinions?

[10], p. 42–51] explains how individuals construct opinion reports in response to the particular stimuli that confront them, introducing the RAS model. The model postulates four axioms: Reception, Resistance, Accessibility, and Response. With the first axiom, Reception, Zaller employs the concept of political awareness to explain that differential levels of cognitive engagement regulate the reception of a message, assuming that certain individuals are more susceptible to information than others. Politically aware people are more selective about the information they internalise, accepting what is more consistent with their values and rejecting inconsistent pieces. Moderately aware individuals are the most susceptible to influence because they care enough to pay attention but lack the resources to resist. Individuals with low levels of political awareness are unlikely to seek out political information, as they do not care enough. However, they are the most vulnerable if they do.

The second axiom, Resistance, focuses on the role of predispositions. Zaller explains that people do not always shift opinions after exposure to political information. Instead, they are affected only if the information contains arguments consistent with their predisposition, since they have the contextual information to perceive a relationship between those and the received messages. Political values are the most relevant predisposition he considers, but race and party attachment are also central.

The third axiom, Accessibility, concerns the concept called consideration, defined as a compound of cognition and affect. Considerations are the reasons that might induce an individual to decide on a political issue. Individuals construct opinion reports in response to the particular stimulus that confronts them, such as the wording of a question or the information they are exposed to. The more recent one consideration has been thought about, the less time it takes to bring it to the top of the head ready for use (Accessibility).

Finally, the Response axiom expands the Accessibility one to explain how people deal with the considerations made salient by certain information or questions to form answers or, more generically, make decisions. Individuals form opinions and make decisions by averaging over a non-random but stochastic sample of relevant considerations, where the sample of considerations may vary between one and large. The probability that individuals will support a given policy depends on the mix of positive and negative considerations available in the person’s mind when answering a question about it.

Considering the RAS model in the theoretical framework of this study, the change in opinion is determined by how the intensity of political information flows interfaces with political awareness and predispositions that ultimately affect opinions. Question 1 focuses on intensity, but, in agreement with the RAS model, political awareness and predisposition create differential patterns of response. Consequently, the second research question is then further specified:

- Q2: Do political awareness and predispositions interface with the differential intensity of political information flows, ultimately affecting the formation of opinions?

## Measuring the intensity of information flows

Intensity is measured with a compound of two established methodologies: Thematic analysis [11] and Shannon entropy [12]. While the former provides a way to classify the content of information flows, the latter provides a way to quantify intensity.

### Thematic analysis

Thematic analysis is a qualitative method to identify themes as patterns within data corpora [11]. “A theme captures something important about the data in relation to the research question, and represents some level of patterned response or meaning within the data set” [, p. 82]. Since this is a qualitative framework, the researcher’s judgement is crucial to determining a theme, and flexibility is recommended.

The data corpus (within the context of this article, called the information flow) was divided into meaningful segments, called messages, labelled with a theme each. For example, if a leader gives a short speech and says “we stand for human rights" and “we will be present at the protest next Saturday", these two segments would be coded as two messages. The first message would be labelled with the theme “human rights" and the second with the theme “activism." Messages can have a single theme, and there can be multiple messages labelled with the same theme. For example, examining the video of a party leader’s speech, if the leader claims “I organise events at my daughter’s school" and “I volunteer at the homeless people shelter every second Saturday of the month", we can code these two segments as two messages, both labelled with the theme “activities for the community". This one theme is repeated twice.

There is a difference between explicit and implicit themes; for example, the way leaders dress, such as a garment with the colours of their party, can appeal to their audience as much as a speech. [, p. 82] captures this difference with the concepts of semantic and latent themes. The former is represented by the semantic meaning of a message and the latter by underlying ideas, assumptions, and conceptualisations, such as the example of a garment. This study considers both.

An information flow operationalised as a data corpus can be composed of one or more messages (each characterised by one theme) selected by researchers according to a criterion relevant to the analysis of the political leader under examination. The thematic analysis of one data corpus will produce two relevant outputs: 1) the number of themes (from 1 to N), and 2) the number of messages labelled with each theme in the data corpus (frequency of the theme ranging from 1 to N).

### Shannon entropy

The number of themes within an information flow and their respective frequency (the number of times a theme appears in an information flow) provide a description of the intensity of the information flow, similar to the definition employed by Zaller and introduced above (the number of media reports covering an issue). Shannon Entropy provides a way to quantify this intensity.

Shannon entropy considers the transmission of messages from a source to an audience and shows that some bits of information are likely to get lost during the transmission. For this reason, if messages are redundant within the transmitted information, there is a much higher probability of being received by the audience compared to a single message transmitted one time only. This is because the latter can get lost in the transmission, not reaching the final destination [12]. Shannon’s work has a focus on physical transmission. Still, it overlaps with communication theory’s concept of redundancy [, p. 10] and the ELM theory [23], both introduced in the Section ‘Intensity of the information flow’. Given the theoretical overlap between these concurring theories, Shannon entropy can be employed as an empirical measure of intensity.

**Fig 1:**
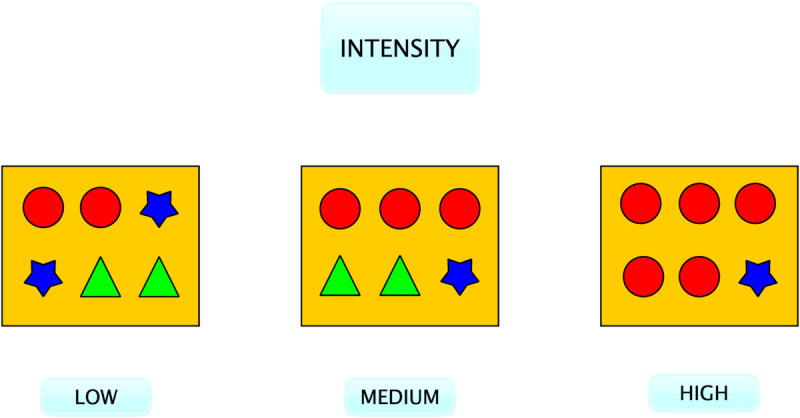
Example of Intensity measured with thematic analysis and Shannon Entropy on three different information flows.

An example can clarify how Shannon entropy is applied to measuring the intensity of information flows. Fig 1 represents the output of three thematic analyses of three different information flows. Each yellow box represents an information flow selected based on relevance, with the objects inside conveying messages that carry specific themes. Each shape represents a different theme. In the example with low intensity, there are three themes in the information flow, each with a frequency of two messages that make them equally likely to be received (two circles, two stars, and two squares). For instance, this could be a leader giving a concise speech on TV that proposes three reasons why people should vote for them and rhetorically repeats each of these three reasons twice. It is equally likely that an individual will receive any of these messages and take them into account when forming opinions. Most likely, the audience will consider one or two of them. Only a very interested audience might pay attention to all of them.

In the case of a medium-level intensity, there are still three themes. However, the one denoted by a circle has a frequency of three messages, and it gets a higher probability of being included in the opinion formation process compared to the triangle that has two messages and to the star that only has one. Keeping the same example, in this case, the leader still mentions three themes but decides to attribute different relevance to each of them. One theme is deemed more important, and the leader decides to use three messages to address it. A second theme has secondary importance, and two messages are enough, while a third one is only covered with one message. Again, a very interested audience might consider the three themes. However, an average audience is more likely to pay attention to the first theme only because it appears more frequently.

In the case of a high intensity, there are only two themes. The one represented by the circle is delivered with five messages, whereas the starred theme is delivered with only one. The red circle has a higher message frequency than the star; therefore, it is very likely that the audience will internalise these messages to form opinions. The political leader, in this case, decides to give full priority to one single theme fully pivotal in their campaign strategy and only briefly mentions a second one that is less important for their communication.

In this example of high intensity, the probability of the audience forming opinions internalising this theme is much higher than in the previous two cases. The intensity of the speech in this third example is much higher than in the previous two. Still, within the example, when leaders encode the speech with low intensity, their purpose is more informative than when they encode the one with high intensity, since they provide a wider array of themes. Conversely, when leaders encode high-intensity speech, their purpose is more persuasive than when they encode one with low intensity.

We can define the number of messages, or frequency, of a theme in an information flow as

$$f_{t}=\sum_{i=1}^{m_{t}}t$$ (1)

where *t* is the theme and *m*_*t*_ is the number of messages labelled with a certain theme within the information flow. The probability of finding each theme in the information flow is

$$P(t)=\frac{f_{t}}{m}$$ (2)

Where m is the total number of messages in the information flow (considering that each message is labelled with one theme, the total number of messages is defined as $m=\sum_{i=1}^{n}f_{t}$). Then, we can use the probability of finding each theme represented by a certain number of messages in an information flow to estimate its intensity using the Shannon entropy. Shannon entropy is defined as

$$E(X)=-\sum_{i=1}^{m}P(x_{t})log_{b}P(x_{t})$$ (3)

where E is the entropy of a discrete random variable *X* with possible values {*x*,...,*x*_*n*_}, and P expresses the probability associated with each possible value [12]. When comparing entropy between two or more information flows, a high number of themes with a low frequency decreases the probability that each theme is considered for opinion formation. This condition corresponds to high entropy and low intensity. On the contrary, a small number of themes with high frequency have small entropy and high intensity. In fact, entropy estimates the chances of losing information during communication [12].

To make the concept of intensity more understandable, we first define the entropy of an information flow as *E*(*F*) and then normalise it by dividing *E*(*F*) by its maximum value. If *P* = 1/*t*, where *t* is the total number of themes in the information flow, the maximum entropy is estimated as $E_{max}=log_{b}(t)$ from which we derive the normalised entropy as

$$E(F{)}_{norm}=\frac{-\sum_{i=1}^{m}P(f_{t})log_{b}P(f_{t})}{log_{b}(t)}$$ (4)

Since we define intensity as the opposite of entropy and the normalised entropy ranges by definition from 0 to 1, we define the intensity of the information flow as

$$I(F)=1-\frac{-\sum_{i=1}^{m}P(f_{t})log_{b}P(f_{t})}{log_{b}(t)}$$ (5)

According to this definition, the maximum intensity of an information flow is 1 and the complete absence of intensity is 0. The empirical range of this measure will be discussed in the remainder of this article.

## Case study and hypotheses

Political leaders have slowly stolen the scene from parties, becoming the centre of political debates [37], of the electoral process [38], and ultimately of the political systems [39]. Studies about leaders have a long tradition in political science, dating back to Max Weber and James Burns [39].

The formation of opinions about political leaders under the reception of new information is relatively unexplored, with some exceptions, such as [40] and [41]. For this reason, the measure of the intensity of an information flow developed in this study is tested on the formation of opinions about a political leader. More specifically, former British prime minister and Conservative leader Theresa May. Mrs. May was appointed for the first time as British prime minister on July 13${\;}^{\text{th}}$, 2016. She did not run for an election, but she took David Cameron’s place as the leader of the Conservative Party and the leader of the country after he was hit by the post-Brexit referendum storm that deeply affected his popularity.

May did not support Brexit, but her leadership came with the burden of pushing it forward. The political and practical difficulties of implementing Brexit threatened to overwhelm the new prime minister [42]. Since May faced the necessity to stay true to her opinion and to respect the will of Britons and her party, her political rhetoric was heavily affected to the point that she was deemed to be absent by her own campaign for the 2017 general election [43] that she called to legitimise her leadership and measure her popularity.

This study was carried out before that election (June 8${\;}^{\text{th}}$, 2017), where May won with a small majority and formed a coalition government. Considering the mixed messages she sent to British voters, focusing on this specific leader provides an opportunity to observe how information affects the formation of opinions about a political leader whose popular support was far from being unquestioned.

In reference to RQ1, two hypotheses can be formulated:

- H1a: Information flows with high intensity affects people’s opinions about Mrs. May [effects]
- H1b: Information flows with low intensity do not affect people’s opinions about Mrs. May [no effects]

In reference to RQ2, the following hypothesis can also be specified.

- H2a: High intensity information flows interface with each proxy of political awareness and predispositions, showing differential patterns of opinion formation about May across subgroups of respondents. [effects]

However, if the intensity of an information flow is low, the reception does not occur, and political awareness and predispositions do not concur with the new information in forming new opinions. In agreement, another hypothesis can also be formulated

- H2b: Low intensity information flows do not interface with each proxy of political awareness and predispositions, showing a homogeneous pattern of opinion formation about May. [no effects]

The research design was pre-registered. The pre-registration is available at the link: https://osf.io/2e6f3/?view_only=71307c58d3584a03879eb0860eb5d52d. However, the terminology employed in the original registration of the hypotheses differs from the current formulation and reflects the point of view of the encoder using the expressions “information with persuasive purposes" for the high intensity and “informative purposes” the low intensity. Moreover, the phrasing of these hypotheses changed from the original registration as well. One statement only captured what this study presents as two hypotheses. Also, for simplicity, political awareness and predispositions were named political attitudes. The registration hypothesises that political attitudes are inversely correlated with influence. When influence (effect of the high intensity information) grows, political awareness and predispositions do not correlate with the outcome variable. This statement translates into the absence of effect hypothesis H2b. As a complementary hypothesis, this study presents H2a as well.

## Methodology

This study employs an online survey experiment with two mock vignettes containing information flows characterised by different levels of intensity: one with high intensity and the other with low intensity. The experimental design received ethical approval from the SSIS College Ethics Committee at the University of Exeter on 20 April 2016 (approval number: 201516–022). The experiment has been designed following [13]. British voters were invited to participate in the study and were prompted with a mock vignette if assigned to one of the two treatment groups. Individuals assigned to the control group were not exposed to a vignette. The experiment was distributed online on the platform *Qualtrics*.

The experiment started on the 18${\;}^{\text{th}}$ of May 2017. Data were collected until 31${\;}^{\text{st}}$, right before the General Election. Participants were not paid and expressed their consent to participate in the study inside the Qualtrics platform before answering the questionnaire.

### Vignettes design

Vignettes are short descriptions of people or situations that can be shown to respondents using surveys to elicit their evaluation of the information they are exposed to. The two vignettes employed in this experiment simulate two information flows and contain pieces of information retrieved online about Theresa May. The two vignettes have the same structure to elicit comparability: each one is composed of one video (2.5 minutes) and two images. Even if the content is not manipulated, combining these three pieces constitutes manipulation, since participants might not have chosen to expose themselves to these information flows.

The manipulation creates one information flow (vignette 1) containing highly intense political information about Theresa May, and one information flow (vignette 2) containing low intense political information about Theresa May. This design used videos and images to make the vignette more effective and engage respondents to a greater extent. Thematic analysis has been performed to identify themes in the narrative (semantic) and visual (latent) narrative. Vignettes do not include campaigning material, both for the higher risk of respondents already being familiar with the content (the experiment went online during pre-election time) and for avoiding negative campaign statements. In fact, negative messages present a higher level of complexity for the formation of an opinion that could have affected the experimental design.

Table 1 summarises the content of the two information flows (vignettes). The total number of messages in both vignettes is very similar to ensure that the two vignettes have the same size. What varies is the number of themes and their respective frequency, in other words, the number of messages attributed to each theme. The intensity is measured in agreement with the definition presented above.

**Table 1:** Mock Vignettes - Manipulation of the information about Theresa May.

Selected Information	Vignette High Intensity (V1)	Vignette Low Intensity (V2)
Piece 1	Video -Conservative Party	Video -BBC
Piece 2	Image -Conservative Party	Image -Wikipedia
Piece 3	Image -Conservative Party	Image -Blog
N of themes	8	17
Tot messages	30	28
Intensity	0.043	0.022

The low intensity vignette reports an intensity of 0.022, and the high intensity vignette has an intensity of 0.043. As explained in the section ‘Measuring the intensity of the information flow,’ the lower the entropy, the higher the intensity.

**Fig 2:**
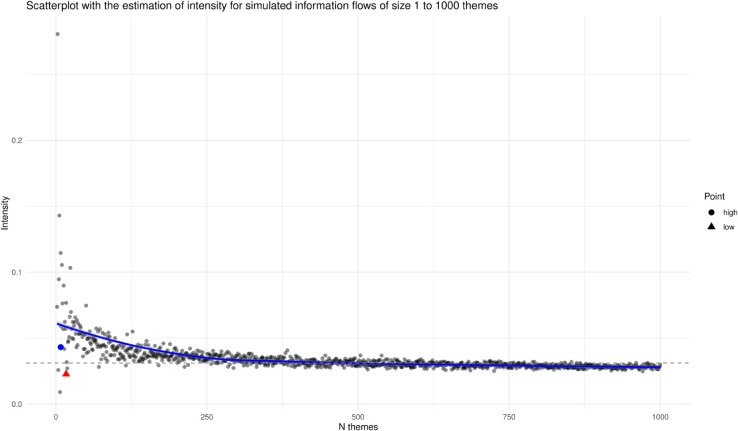
Simulated experiments to place the intensity of the two vignettes into an empirical context. Scatterplot showing the behaviour of the intensity measure with 1000 simulated information flows with a number of themes from 1 to 1000 each. Dashed horizontal line denotes the mean of the intensity.

**Fig 3:**
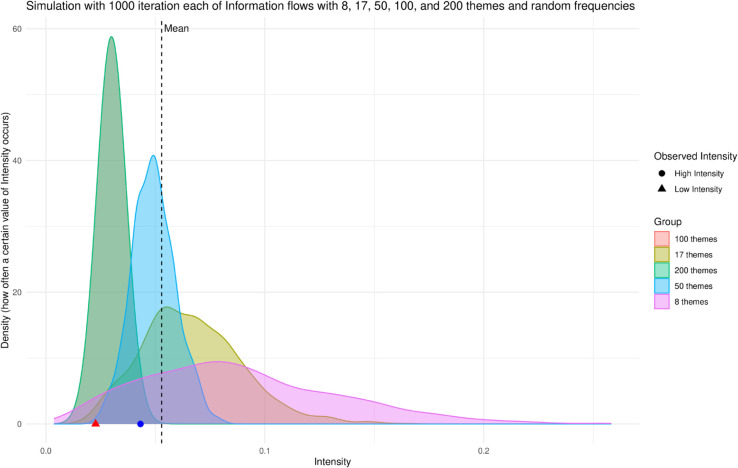
Simulated experiments to place the intensity of the two vignettes into an empirical context. Density plot showing five different experimental simulations, each with 1000 runs, respectively with 8 themes (such as vignette 1), 17 themes (such as vignette 2), 50 themes, 100 themes, and 200 themes.

We can appraise that, as expected, the low intensity vignette has a lower intensity than the high intensity one. However, both scores are really small and, by themselves, are hard to interpret. To place these scores in context, we compare the empirical scores estimated from the two vignettes with simulated scores that create a baseline. Figs 2 and 3 present the results of simulations that estimate the intensity in a selection of synthetically reconstructed information flows. Fig 2 displays the results of a simulation of 1000 information flows that range, respectively, from 1 to 1000 themes with a random number of messages. On the x-axis, we observe the number of themes, while on the y-axis, we observe the measured intensity for each simulated information flow. From the figure, we can see that random information flows characterised by a small number of themes tend to have higher values of intensity, and random information flows with a large number of themes tend to show smaller values of intensity. This result is not surprising, as it reflects the definition of Shannon entropy.

The mean intensity is marked on the plot with a horizontal dashed line and has a value of 0.034. The intensity of the two vignettes used in this study is displayed in the plot for comparison. The intensity of the vignette to which we refer as high intensity shows a score above the mean (blue circle in the plot). In contrast, the vignette that we refer to as low intensity has a score below the mean (red triangle).

Fig 3 displays the results of the estimated intensity in 5,000 information flows divided into five groups. Each group contains 1000 simulations of information flows, respectively, with 8, 17, 50, 100, and 200 themes. The number of themes 8 and 17 are considered since they are respectively the number of themes of vignettes 1 and 2. The mean intensity across these 5,000 simulations is 0.052 (dashed vertical line in the plot). In the plot, we can see that the vignette referred to as high intensity has an intensity close to the mean (represented by the blue circle). In contrast, the vignette referred to as low intensity has an intensity that is far lower than the mean (red triangle).

With these simulations, we can place the intensity of the vignette in a range and claim that vignette 1 has a much higher intensity than vignette 2, considering the simulated range. Future work will develop an empirical range using more simulated data and real data to enhance the baseline presented in this study. This task goes beyond the scope of the present study. Considering that the high intensity vignette did not employ campaign material, it makes sense that its intensity is around the mean of the simulations’ intensity and not higher. Considering that the low intensity vignette was not designed to be intense, its placement in comparison to the simulated mean seems in line with the theory. In the supporting information S1 Table and S1 Appendix provide details on the vignette construction (themes, number of messages expressing their frequencies, and coding procedure). Vignettes were pre-tested on a cohort of students.

### Survey design

The effects of the vignettes are measured with eight statements that respondents evaluate on an eleven-point Likert scale. The statements were designed to capture the themes in the two information flows. The survey also employed established proxies of political awareness, such as Interest in Politics, Political Knowledge, and Time Spent Online Looking for Political Information. The selected proxies for predispositions are Trust in British Politicians, Satisfaction with British Democracy, and Party affiliation. The questions were reproduced from the British Election Study Survey [44]. The complete list of questions is in the supporting information S2 Appendix.

### Sampling and randomization

The experiment collected a convenience sample of 1,159 British respondents over 18 years old who replied to the online survey. The employment of convenience samples is increasingly common in survey experiments [45,46]. The respondents were recruited within the framework of the project that partially financed this study. The dataset is available at the link https://osf.io/cb3yt/?view_only=18ec88839e0c44a697509332ffd7bf50. Power calculation was used for the experimental design: for a power of 0.8 and a 5% error to detect a 0.5 variation on the 0-10 scales of each statement, the achievement of significance can be estimated with a minimum of 630 respondents evenly distributed in the three groups.

Table 2 summarises the experimental design and the randomisation of the respondents. Qualtrics randomly subdivided the sample into three groups. Participants randomly assigned to one of the treatment groups were exposed to one of the two vignettes and asked to complete the survey. The control group was only exposed to the survey. The activity required ten minutes for the treated participants and around seven for those assigned to the control.

**Table 2:** Experimental design.

	Groups		
Experiment Components	Vignette 1 (V1)	Vignette 2 (V2)	Control (V0)
Vignettes	High Intensity	Low Intensity	No Vignette
Survey	Entire Survey	Entire Survey	Entire Survey
N Respondents	406	340	413

### Variables

A principal component analysis (PCA) [48] is run on eight statements’ evaluations that measured the effects of the vignettes (details in the supporting information S1 Fig.). The PCA measured two latent constructs. One construct loads on the questions measuring Theresa May’s political performance; the other captures the evaluation of her personal capability. The first latent construct is named *performance evaluation* and the second *capability evaluation*. These two constructs are analysed as outcome variables and used to assess whether the vignettes affected the respondents’ evaluations of the statements about Theresa May.

Data about political knowledge constitutes a knowledge scale [49]. Respondents’ political orientation was measured with a battery of Propensity To Vote (PTV) questions [47]. PTV questions are recoded into a categorical variable named party affiliation (1. respondents that did not score any party above six as a “protest", 2. respondents that expressed the highest score above six for a specific party as supporters of that party, 3. respondents expressing the same two highest scores above six for two different parties at the same time as “undecided".).

### Experimental cases

The effects of two vignettes with high and low intensity are observed using the same experimental design, but treated as two separate experiments. The experimental design comprises two experiments, since the effects of one vignette are independent of the other, and this study focuses on comparing these two situations. Accordingly, the data is analysed in two different experiments.

It is also important to distinguish between the two latent constructs we measured with the PCA, since they tap into different sets of opinions (two different topics) formed in reaction to the exposure to the vignettes. Therefore, considering two experiments and two constructs, the collected data constitute four cases of these two experiments that we summarise in Table 3. The Table introduces the two experiments and the four cases that are analysed. The battery of variables used to estimate the effect of each of the two experiments is processed with a PCA. The PCA outputs two constructs, thematically labelled as ‘performance’ and ‘capability’. The design produces four interpretable cases out of two experiments. Thus, each hypothesis is tested on the four different cases that this study examines. For each of the cases, the group treated with one of the two vignettes is compared to a control group.

**Table 3:** Summary of the two experiments analysed as four cases, given that the PCA outputted two components.

Experiment	Cases	Components (Outcome)	Treatments	Label
High Intensity	Case 1	Performance	Vignette (1)	C1
	Case 2	Capability	Vignette (1)	C2
Low Intensity	Case 3	Performance	Vignette (2)	C3
	Case 4	Capability	Vignette (2)	C4

### Models specification

Hypotheses 1a and 1b are tested against each of the four cases by the estimation of the average treatment effect (ATE) [, chapter 8] computed with OLS. Hypotheses 2a and 2b are tested against each of the four cases with the estimation of the Conditional Average Treatment Effect (CATE) and by observing the variance between the CATE and the ATE of each variable [, chapter 10]. The CATE conditions the estimations of the ATE on a subgroup of respondents specified by one or more chosen covariates. It is estimated using interaction effects on OLS, where each variable of interest is specified as the interaction effect in the model.

Since Zaller’s RAS model [10] implies that subgroups of respondents characterised by different levels of political awareness and predispositions might deviate from the overall effect in the sample when exposed to information, by looking at the variance between the CATE for each covariate and the ATE for the overall sample, we can identify the indicators characterising subgroups of respondents that differ from the mean in processing information (absence of constant effects). The CATE is estimated separately for each covariate to map the unbiased pattern of interactions. CATE is also estimated using OLS, with one interaction term per model.

The variance between ATE and the CATE for each indicator is computed using Anova, as it enables us to observe the effect of each covariate and test against the null hypothesis of constant effects (ATE). CATE analysis is similar to considering variables as moderators, but it involves theoretical differences between the concepts of moderation and effects that are conditionally valid for subgroups of respondents only. The discussion of this point goes beyond the scope of this study. The R code employed to estimate the models is available in the supporting information S3 Appendix.

## Description of the sample

In the convenience sample of 1,159 respondents, men and older people are over-represented (67.04% of the sample is men, and 48.05% is over 50). The majority of participants are highly educated (41.76% with a University degree), 71.52% own their home outright or have a mortgage, which qualifies this population as more affluent than the average (details in the supporting information S2 Table). The sample is evenly distributed among the three groups (details in the supporting information S3 Table). The indicators of political awareness measured very high average scores with 4.42 out of 5 for interest in politics, 10.79 out of 12 for political knowledge, and 3.38 out of 5 for time spent online looking for political information. Comparing the means and standard deviations across groups, the differences are irrelevant (details in the supporting information S4 Table). In relation to the proxies for predispositions, Trust in British politicians measures 2.45 out of 5, and satisfaction with British democracy 2.44 out of 5, showing an average level of trust and satisfaction. The same trend is also shown in the mean and standard deviation by the group. Respondents’ political orientation was measured with a battery of Propensity To Vote (PTV) questions [47]. On a scale from 0 to 10, the Labour Party scored the highest (mean 5.55, s.d. 3.83), followed by the Liberal Democrats (mean 4.52, s.d. 3.45), the Green Party (mean 4.36, s.d. 3.45), the Conservative Party (mean 3.33, s.d. 4.02), Plaid Cymru (Mean 2.09, s.d. 3.22), Scottish National Party -SNP (mean 1.80, sd.3.14) and the United Kingdom Independence Party -UKIP (mean 1.19, s.d. 2.66). In general, respondents in this convenience sample are more likely to vote for left-wing parties. Political preferences are evenly distributed across groups (more details are available in the supporting information S5 Table).

## Results

### The effects of differentially intense information

Table 4 displays the results of the ATE estimated with an OLS (in the supporting information S6 Table and S2 Fig. provide further information on the analysis; models that include covariates are also provided in the supporting information S3 Fig., showing no significant variation from the results reported here). C2 (Capability - High Intensity) shows the most interesting result, since the probability that what we observe happened by chance is very small (*p* < 0.001), and the effect is quite strong (0.48). In C2, the high intensity vignette affected the respondents, increasing the mean evaluation of Theresa May’s capability, considering the entire group of respondents as theorised, showing evidence for hypothesis H1a.

**Table 4:** ATE computed by OLS –baseline models. Table produced using texreg [50]

	C4	C3	C2	C1
ATE	–0.12	0.02	0.48^***^	0.06
	(0.07)	(0.07)	(0.07)	(0.07)
R^2^	0.00	0.00	0.06	0.00
Adj. R^2^	0.00	–0.00	0.06	–0.00
Num. obs.	753	753	819	819

^***^p < 0.001; ^**^p < 0.01; ^*^p < 0.05

C3 (Performance - Low Intensity) and C4 (Capability - Low Intensity) also show evidence in favour of the expectations stated with the hypotheses. The respondents treated with the low intensity vignette do not appear to have an opinion any different from the respondents in the control group, both for the evaluation of the capability and performance of Theresa May. The p-value is not statistically significant, and the effect size is very small. The results of C3 and C4 both support hypothesis H1b since exposure to low intensity political information is not expected to affect opinions. The results we observe in C1 (Performance - High Intensity) do not show evidence in favour of hypothesis H1a. The vignette with high intensity did not affect the respondents’ evaluation of Theresa May’s performance. The p-value is not significant, and the effect size is very small.

There is no imbalance [51] between the group treated with the high intensity vignette and the control, nor between the group treated with the low intensity vignette and the control. The covariate imbalance analyses are reported in the supporting material S7 Table and S8 Table.

### The effects of differentially intense information on subgroups of respondents

After computing the CATE for each indicator of political awareness and predispositions, Anova computes the variance and shows the difference between the ATE and the CATE. ATE is computed as the baseline models since the analysis in the previous sections showed robustness between fully specified models and baseline ones. CATE scores are computed with OLS using an ATE baseline models with one interaction variable per time. The variance between ATE and CATE assesses whether subgroups defined by the covariates are subject to different effects than the average population. Table 5 presents the results of the Anova analysis, including F-statistics and p-values for each covariate used as a proxy for political awareness and predisposition (blocks at the top and the centre). There is a third block (bottom) that presents results for demographics. The results are separately reported for each of the four cases. F-statistics expresses the standardised difference between the CATE and the ATE; the p-value informs us of its statistical significance. The significant coefficients denote those covariates that characterise subsets of respondents deviating from the average effect generated on subgroups of respondents by the vignettes.

**Table 5:** The table displays F-statistics, and P-value computed with Anova to show the standardised variance between ATE and CATE for each covariate ^***^p < 0.001; ^**^p < 0.01; ^*^p < 0.05.

Variable	C1 Performance	C2 Capability	C3 Performance	C4 Capability
	High Intensity	High Intensity	Low Intensity	Low Intensity
Interest in Politics	3.1	4.013*	4.013	3.426
Knowledge	0.349	2.675	5.293*	0.817
Time Online	3.744	14.961***	3.002	1.051
Satisfaction	4.27*	124.388***	0.033	1.741
Trust	5.249*	52.887***	0.281	0.519
Party Affiliation	1.453	21.63***	0.345	1.058
Gender	0.027	0.025	1.12	0.097
Age	0	3.082	0.302	2.353
Education	0.088	0.998	0.595	0.006
Housing	0.124	2.594	0.155	0.123

C2 (high intensity vignette and the capability evaluation) shows the most interesting results once more. For the political awareness proxies, we observe significant F-statistics for interest in politics (4.013, *p* < 0.05) and time online (14.961, *p* < 0.001). The three proxies that measure predispositions are all showing significant variances from the mean of the complete sample, with satisfaction with the British democracy (124.388, *p* < 0.001), trust in British politicians (52.887, *p* < 0.001), and party affiliation (21.63, *p* < 0.001). C2 supports hypothesis H2a and Zaller’s RAS model since the significant proxies show different effects for those subgroups in the sample.

The results of C3 and C4 refer to the effect of the low intensity vignette, respectively, on the evaluation of capability and performance. They show no difference between the ATE and the CATE for every covariate, except political knowledge in the C3 case (evaluation of May’s performance), which constitutes an exception with a p-value inferior to 0.05. In the supporting information S4 Fig. presents a boxplot with the difference in mean between the control group and the group treated with the vignette conditioned to knowledge for C3. The plot indicates that categories 0 to 3 and 5 of knowledge have no respondents in either the treated or control group. This result, rather than an effect of the vignette, is a bias of the data that is skewed towards high levels of knowledge, leaving empty categories at low levels. These findings show evidence in favour of hypothesis H2b.

The results displayed for C1 refer to the respondents treated with the high intensity vignette and assessed against the evaluation of May’s performance. The two covariates measuring trust in British politicians and satisfaction with British democracy are significant with a p-value inferior to 0.05, denoting that even if the overall sample was not affected, the subgroups of individuals analysed with these two variables are. These two scores show evidence in favour of H2a. In the supporting information S5 Fig. reports boxplots showing the effects on respondents with different levels of trust and satisfaction. The effect is strong for respondents who show high levels and almost absent for those who show lower levels. Results do not show significant variances between the ATE and the CATE for the demographics, proving that they do not affect the reception of information. These results provide further evidence of the absence of an imbalance.

## Discussion

Opinion change follows specific patterns. [4] explains three of them: 1) the *activation* of an already existing latent attitude that generates a new opinion; 2) the *conversion* from one opinion to another –that involves an attitude change; 3) the *reinforcement* of an existing opinion –cognitive consistency. According to [10], the receiver can resist an information flow in three ways: 1) *Partisan Resistance*, where the receivers can recognize the received information flows as inconsistent with their attitudes and therefore reject it accordingly; 2) *Inertial Resistance*, when the receiver already possesses a large body of considerations and the new information flow produces new considerations that are swamped by the effects of the pre-existing ones; 3) *Countervalent Resistance*, where receivers can resist the new information providing counter-arguments in accordance to their pre-existing knowledge.

Considering this established framework, C1 (May’s performance, high intensity) can be interpreted as partisan resistance to the high intensity information flow. Most likely, every respondent already had an opinion about the performance of the Prime Minister and was not willing to change this opinion after exposure to the vignette used for this study. Only the very satisfied and trustworthy respondents let the information flow affect their opinions. The behaviour of these respondents can be explained by opinion reinforcement.

As concerns C2 (High Intensity - Capability), we observed an opinion change that can be attributed to Activation. Considering that the majority of respondents did not support the Conservative Party (supporting information S5 Table), it is crucial to observe that the high intensity vignette shifted the evaluation concerning May’s capability in a significant way. This result should be considered a case of Activation. Labour supporters, after being exposed to the treatment, “activated” the consideration that May can be capable even if they do not like her as a political leader and formed opinions measured with the battery of questions. Even if these respondents were not May’s supporters and did not approve of her political agenda, they had no previous firm belief concerning May’s capability. The treatment –consistently depicting former Prime Minister May as capable– activated an attitude that was not there before. Even if they still did not support May as a politician, after exposure to the treatment, they formed a “parallel" opinion about her capabilities. This parallel opinion does not alter their overall view of the former Prime Minister; instead, it highlights an aspect previously overlooked, an attitude that was triggered by the treatment. In reference to the conservative respondents, we measured a reinforcement of the opinion they already had about the capability of Theresa May after exposure to the intense information flow.

The experimental case that assesses the effect of the low intensity vignette on May’s performance (C3) can be interpreted as Countervalent Resistance. The information flow provided by the treatment was not intense enough to affect opinion formation. The respondents, most likely, already had an opinion about May’s performance and were able to counterargue with their already existing opinions.

Finally, the experimental case assessing the effect of the low intensity vignette on May’s capability can be considered a case of inertial resistance. While most respondents likely had an opinion about May’s performance, they probably did not have a strong opinion about her capability. Still, the information flow they were exposed to was not intense enough to have an effect. Hence, the volume of pre-existing knowledge swamped the information related to May’s capability, and the information flow was disregarded due to inertia.

In light of these considerations, the measurement of intensity using thematic analysis and Shannon entropy shows results coherent with the literature on the topic. This coherence provides validity for the measure introduced in this study.

Although the results align with the theory, it is necessary to consider that the experiment sampled a non-random population, which was predominantly highly educated, male, and with a left-wing political orientation. Further experiments are needed to empirically observe the effects on different groups of people and corroborate the findings of this study. At the same time, future work should establish an empirical scale for the intensity measure.

This study finds that a level of intensity close to the mean of simulated information flows is able to affect the opinion of the respondents. Still, we cannot determine at this stage whether a mean value on an intensity scale is sufficient to influence opinion in other cases. More simulated studies and experiments with real respondents are needed to follow up and answer these questions. This goal extends beyond the aim of this study. Introducing the measure and testing established hypotheses in political communication is the contribution that this article offers.

## Conclusion

This study introduced a methodology to measure the intensity of political information flows. It showed that more intense information flows are more likely to affect opinions than those with low intensity. Moreover, low levels of political awareness and partisan political considerations, such as trust, satisfaction, and party affiliation, drive information reception and make opinion shifts more likely to happen after exposure to intense information flows. These results are in line with the RAS model [10].

The two experiments, analysed as four cases, showed different results. The vignette with high intensity did not affect the evaluation of Theresa May’s performance (C1) for most respondents. Only the audience characterised by a high level of satisfaction with British democracy and those with high trust in British politicians were affected by the exposure to the high intensity vignette. Even if there are no average effects across the entire sample, there are effects for those respondents who show these specific characteristics. This effect can be interpreted as partisan resistance for the majority of the sample and as opinion reinforcement for the respondents characterised by high trust and satisfaction.

The same vignette affected the evaluation of Theresa May’s capability (C2) on average for the complete sample. Also, we reported more substantial effects for respondents with a moderate interest in politics, those who spend less time online looking for political information, and those who are more satisfied with the government and British politicians (same as case one). These findings are in line with the RAS model [10]. This second case can be explained as opinion activation [4]. In fact, even if the same respondents might have a negative opinion about May’s performance, they might not have an opinion at all about May’s capability since this issue is less crucial and less prevalent in the media. Hence, it was more straightforward to activate an opinion about it.

The vignette with low intensity did not affect either the evaluation of performance (C3) or the capability (C4). The absence of effects is homogeneous for the entire sample, including the sub-samples based on every respondent’s characteristics. The former case is explained by countervalent resistance since respondents already had an opinion concerning May’s performance. The latter case is explained by inertial resistance since respondents might not have had an opinion about May’s capability, and the vignette was not intense enough to activate one.

Further research is needed to explore the validity and reliability of the measure of intensity introduced in this study. Still, these results suggest that information flow intensity is a robust way to predict the effects of information on opinion change. Also, previous theory on the subject is in line with the results displayed by this measure, showing evidence for the validity of the measure.

## Supporting information

S1 Table Number of themes and frequencies in each vignette. (PDF)

S1 Appendix Content of the two Vignettes. (PDF)

S2 Appendix Survey Design. (PDF)

S2 Table Descriptive statistics of the entire sample. (PDF)

S3 Table Descriptive statistics by group. (PDF)

S4 Table Descriptive statistics Political Awareness and Predispositions. (PDF)

S5 Table Descriptive statistics Party Affiliation. (PDF)

S1 Fig Scree Plot Principal Component Analysis. (PDF)

S6 Table Average Treatment Effect (ATE) computed with t-test. (PDF)

S2 Fig Average Treatment Effect (ATE) results visualized. (PDF)

S3 Fig Average Treatment Effect Analysis with covariates. (PDF)

S7 Table Imbalance Analysis for the High Intensity treatment. (PDF)

S8 Table Imbalance Analysis for the Low Intensity treatment. (PDF)

S4 Fig Difference in control and treatment group in interaction with Knowledge -case3. (PDF)

S5 Fig Difference in control and treatment group in interaction with Satisfaction and Trust -case1. (PDF)

S3 Appendix R Code. (PDF)
