# Supplementary material for: Measuring the effects of differentially intense information on political opinions
Source: PLoS One. 2025 Nov 26;20(11):e0333129. doi: 10.1371/journal.pone.0333129 (PMC12654871; doi:10.1371/journal.pone.0333129)
Supplement: S1 Table — (PDF) [file pone.0333129.s001.pdf]

S1 Table: Number of themes and frequencies in each vignette

Themes in the vignettes	**High intensity** Frequency (N messages)	**Low intensity** Frequency (N messages)
1. May's Skills	6	2
2. "Country that works for everyone"	2	2
3. "Make Britain Fairer"	5	1
4. May's Competence	5	2
5. Managing Difficult Periods	2	1
6. May is Trustworthy	5	2
7. May is Moral	3	1
8. May is one of us	2	1
9. Successful Woman/Fashionista	missing	1
10. May's role in the evolution of the Conservative Party	missing	2
11. Evolution of the Conservative Party	missing	2
12. New Conservative ideology perceived by the audience	missing	3
13. Re-branding of the Conservative Party	missing	2
14. Disagreement with the slogan "Country that works for Everyone"	missing	2
15. Skepticism on May's political strategies	missing	2
16. Ideology Appealing Everybody	missing	1
17. Conservative Convention	missing	1
Entropy	1.99	2.77

Table 1: Themes, their frequencies (N messages) and level of entropy within the two vignette treatments.
