# Supplementary material for: Measuring the effects of differentially intense information on political opinions
Source: PLoS One. 2025 Nov 26;20(11):e0333129. doi: 10.1371/journal.pone.0333129 (PMC12654871; doi:10.1371/journal.pone.0333129)
Supplement: S1 Appendix — (PDF) [file pone.0333129.s002.pdf]

S1 Appendix: Content of the Vignettes

Supporting Information –Vignettes preparation

The vignettes employed in the online experiment were prepared by selecting two homogeneous sets of pieces of information from the web and identifying common themes in the two sets using thematic analysis (Braun and Clarke, 2006). Keeping the same length and structure in the two treatments, one is characterized by high intensity (several messages carrying a small number of themes); the other is characterized by low intensity (higher number of themes, each carried by a relatively small number of messages). The thematic analysis of the full data corpus employed for the vignette is available in the following sections.

Vignette number one (Low Entropy - High Intensity)

The first vignette is composed of three pieces of information selected for the creation of high intensity content. The first piece of information is a 2:40 minutes video posted by the Conservative party on their YouTube channel on the 5th of October 2016 (Conservative Party, 2016). The second piece of information composing the first vignette is a screenshot taken from Theresa May's website (May, ndb) that is no longer available online since the website was modified just before the *Snap* election. The third piece of information composing this vignette is another screenshot taken from the picture gallery of May's website (also no longer available).

Thematic Analysis vignette 1 - High Intensity.

Themes:

1. Skills
   (a) video (latent): May is the narrator, and she shows self-confidence and appropriateness
   (b) video (semantic): May explains her vision and her plans with determination
   (c) video (latent): May looks smart and proactive when suggesting solutions for a series of concerns
   (d) video (latent): May looks cheerful, energetic and enthusiastic when meeting people
   (e) picture 1 (latent): May walks forward showing self-confidence
   (f) picture 2 (latent): May is perfectly comfortable within the three contexts
2. Country that works for everyone
   (a) video (semantic): slogan presented
   (b) picture 1 (semantic): slogan presented
3. Make Britain Fairer
   (a) video (semantic): slogan presented
   (b) video (semantic): May claims Britain is a great meritocracy, and talent is the only thing that gets people on in life. Thus, she provides equal opportunities for everyone independently from ethnicity, sexual orientation, and social class
   (c) video (latent): shows images of people belonging to different ethnicities
   (d) picture 1 (semantic): fairness for the Britons and not only for Europe
   (e) picture 2 (latent): May equally appreciate different kinds of people
4. Competence
   (a) video (semantic): May shows she has a plan for the country
   (b) video (latent): May is with her people taking care of the constituency's problems
   (c) video (semantic): as a narrating voice May provides information about the political and economic state of the country

(d) picture 1 (latent): May shows she has a plan for the country
(e) picture 2 (latent): May shows she can take care of pupils and elderly people

5. Managing Difficult Periods
   (a) video (semantic): May claims she will get a Brexit good deal for the Britons
   (b) picture 1 (semantic): May suggests she has a solution for the Brexit
6. Trustworthy
   (a) video (latent): May says she believes in her country and her community by stating that it is strong
   (b) video (latent): May says she will give people what they voted for and appears in several images with some of them
   (c) video (latent): May appears in a series of images successfully dealing with political matters
   (d) picture 1 (latent): the combination of self-confidence, a clear plan, a smile and a way of dressing appropriate to her role, shows she can be trusted
   (e) picture 2 (latent): May shows that she can be trusted since she is taking care of pupils and elderly people
7. Moral
   (a) video (semantic): May claims she stands for who feels less strong (a reference to fairness)
   (b) video (latent): May is shown as a baby with her parents (a reference to family)
   (c) picture 2 (latent): May shows interest for pupils and elderly people (a reference to compassion)
8. May is one of us
   (a) video (latent): May appears with normal people in her constituency
   (b) picture 2 (latent): May behaves like a normal person enjoying time with elderly people and pupils

Vignette number two (High Entropy - Low Intensity)

The second vignette is composed of three pieces of information selected for the creation of low intensity content. The first piece of information that composes the low intensity vignette is a 2:53 minutes video from the BBC aired on the 4th of October 2016 (Newsnight BBC, 2016). A screenshot from a blog, showing Theresa May walking, constitutes the second piece of information that composes the high entropy treatment. The last piece of information that forms the high entropy treatment is a screenshot of the Wikipedia page dedicated to Theresa May (May, nda).

Thematic Analysis vignette 2 - Low Intensity.

Themes:

1. Skills
   (a) picture 1 (latent): May looks self confident
   (b) picture 2 (latent): It is shown May achieved many goals
2. Country that works for everyone
   (a) video (semantic): slogan presented
   (b) video (semantic): One person interviewed during the Conservative convention claims the Conservatives has always been the party that works for everyone
3. Make Britain Fairer
   (a) video (semantic): One person interviewed during the Conservative convention claims the Conservatives is the party for the working men
4. Competence
   (a) video (latent): May looks competent addressing the Conservative party members
   (b) picture 2 (latent): May looks competent since she had several public roles
5. Managing Difficult Periods
   (a) video (latent): May wants to change the negative attitude that the audience showed toward the Conservatives when they called them the nasty party
6. Trustworthy
   (a) picture 1 (latent): May looks professional and dynamic, able to achieve goals
   (b) picture 2 (latent): May's achievements show she can deliver what she plans to do
7. Moral
   (a) video (latent): May is shown in an image in the company of her husband (reference to family)
8. May is one of us
   (a) picture 1 (latent): May looks like a normal woman in a working day
9. Successful Woman/Fashionista
   (a) picture 1 (latent): This image is part of a collection and this context suggests that she was photographed because of her role and outfit
10. May's role in the evolution of the Conservative Party
    (a) video (latent): May is presented in the story-line as the one that pushed the party forward. She says "they called us the nasty party"

   (b) video (latent): May is presented as the one person renewing the ideology of the party by making it more inclusive

11. Evolution of the Conservative Party

    (a) video (semantic): David Cameron's role. He says "I was the future once"

    (b) video (semantic): Speaker telling the story of the Conservative party

12. New Conservative ideology perceived by the audience

    (a) video (semantic): One person in the audience says that the new Conservative combine pragmatism with social concerns

    (b) video (semantic): One person in the audience says that the new Conservative are holding the center and the right wing voters at the same time

    (c) video (semantic): One person in the audience says that the new Conservative slogan is populist

13. Re-branding of the Conservative Party

    (a) video (latent): the new logo is shown

    (b) video (semantic): Speaker telling the story of the new branding strategy

14. Disagreement with the slogan "Country that works for Everyone"

    (a) video (semantic): One person in the audience says that the new Conservative slogan is rather vague

    (b) video (semantic): Speaker says with sarcasm that maybe the Conservative party will soon be working for everyone

15. Skepticism on May's political strategies

    (a) video (semantic): One person in the audience says that she is not the right person for being in power

    (b) video (semantic): Speaker says he does not believe in May's political strategies

16. Ideology Appealing Everybody

    (a) video (semantics): Speaker says that the new Conservative ideology is trying to be appealing for everyone and this is the dream of every politician

17. Conservative Convention

    (a) video (latent): Several images of the Convention location show how it is well organized and efficient
