# Supplementary material for: Measuring the effects of differentially intense information on political opinions
Source: PLoS One. 2025 Nov 26;20(11):e0333129. doi: 10.1371/journal.pone.0333129 (PMC12654871; doi:10.1371/journal.pone.0333129)
Supplement: S2 Appendix — (PDF) [file pone.0333129.s003.pdf]

S2 Appendix: Survey design

- Statements to evaluate
    1. Theresa May is a skilled politician
    2. Theresa May is the right person for managing a difficult period
    3. Theresa May is a trustworthy MP
    4. Theresa May has no moral
    5. Theresa May is the right person to lead a country that works for everyone
    6. Theresa May will not make Great Britain fairer
    7. Theresa May is one of us
    8. Theresa May is a competent Prime Minister
- Interest in politics
    1. How interested would you say you are in politics? (5 point Likert scale)
- Political knowledge (statement to rate: “True”, “False”, “I don’t know”) as measured in the British Election Study survey.
    1. Polling stations close at 10.00 pm on election day.
    2. No-one may stand for parliament unless they pay a deposit
    3. Only taxpayers are allowed to vote in a general election
    4. The Liberal Democrats favour a system of proportional representation for Westminster elections
    5. MPs from different parties are on parliamentary committees
- Time Online spent looking for political information (5 point Likert scale)
    1. On an average day, how much time do you spend using the Internet for news about politics and current affairs?
- Trust in British politicians (5 point Likert scale)
    1. I generally trust British Politicians
- Satisfaction with the British Democracy
    1. On the whole, I’m satisfied with the way democracy works in this country.
- Party Affiliation (11 point Likert Scale)
    1. “How likely is it that you would ever vote for each of the following parties?” (followed by list of parties)
