# Supplementary material for: Measuring the effects of differentially intense information on political opinions
Source: PLoS One. 2025 Nov 26;20(11):e0333129. doi: 10.1371/journal.pone.0333129 (PMC12654871; doi:10.1371/journal.pone.0333129)
Supplement: S2 Table — (PDF) [file pone.0333129.s004.pdf]

S2 Table

	Demographics N = 1159		
Variable	Categories	Frequency	Respondent %
Gender	Male	777	67.04 %
	Female	377	32.53 %
	Other	5	0.43 %
Age	18-35	332	28.64 %
	36-50	270	23.3 %
	50+	557	48.05 %
Education	Post-Graduate	311	26.83 %
	First Degree	484	41.76 %
	A-level or equiv.	239	20.62 %
	GCSC or equiv.	93	8.02 %
	No formal qualif.	32	2.76 %
Housing Status	Own Home outright	421	36.32 %
	Own home on mortgage	408	35.20 %
	Rented from local authority	31	2.67 %
	Rented from private landlord	231	19.93 %
	It belongs to housing association	37	3.19 %
	I don't know	31	2.67 %

Table 2: Descriptive statistics - complete sample
