# Supplementary material for: Measuring the effects of differentially intense information on political opinions
Source: PLoS One. 2025 Nov 26;20(11):e0333129. doi: 10.1371/journal.pone.0333129 (PMC12654871; doi:10.1371/journal.pone.0333129)
Supplement: S3 Table — (PDF) [file pone.0333129.s005.pdf]

S3 Table: Descriptive statistics by group

% Differences in Groups				
Variable	Categories	Vignette 1<br>N = 406	Vignette 2<br>N = 340	Control<br>N = 413
Gender	Male	-0.4	-0.28	0.27
	Female	0.22	-0.18	-0.09
	Other	-0.19	0.45	-0.19
Age	18-35	-0.32	-1	1.14
	36-50	1.08	0.22	-1.27
	50+	-0.76	0.77	0.13
Education	Post-Graduate	-2.2	2.29	0.29
	First Degree	-0.14	-5.58	4.73
	A-level or equiv.	2.53	-1.51	-1.25
	GCSC or equiv.	0.1	2.86	-2.46
	No formal qualif.	-0.3	1.96	-1.31
Housing Status	Own Home outright	0.64	-1.62	0.72
	Own home on mortgage	-1.46	0.7	-0.24
	Rented from local authority	-0.21	0.56	-0.25
	Rented from private landlord	0.51	-1.41	0.65
	It belongs to housing association	0.5	0.63	-1.01
	I don't know	0.03	1.15	-0.98

Table 3: Descriptive statistics - by Groups
