# Supplementary material for: Measuring the effects of differentially intense information on political opinions
Source: PLoS One. 2025 Nov 26;20(11):e0333129. doi: 10.1371/journal.pone.0333129 (PMC12654871; doi:10.1371/journal.pone.0333129)
Supplement: S4 Table — (PDF) [file pone.0333129.s006.pdf]

S4 Table: Descriptive statistics Political Awareness and Predispositions

Group	Statistics	Int. in Pol.	Knowledge	Time Online	Trust	Satisfaction
Total	μ	4.42	10.79	3.38	2.45	2.44
	σ	0.71	2.16	0.99	1.08	1.31
High Intensity	μ	4.41	10.68	3.37	2.43	2.51
	σ	0.66	2.28	1.02	1.08	1.30
Low Intensity	μ	4.45	10.81	3.42	2.48	2.39
	σ	0.70	2.10	0.96	1.10	1.34
Control	μ	4.39	10.89	3.36	2.46	2.39
	σ	0.76	2.07	0.99	1.06	1.27

Table 4: Descriptive Statistics Political Awareness and Predispositions by group
