# Supplementary material for: Measuring the effects of differentially intense information on political opinions
Source: PLoS One. 2025 Nov 26;20(11):e0333129. doi: 10.1371/journal.pone.0333129 (PMC12654871; doi:10.1371/journal.pone.0333129)
Supplement: S5 Table — (PDF) [file pone.0333129.s007.pdf]

S 5 Table: Descriptive statistics Party Affiliation

Descriptive Statistics of the Party Affiliation								
		Cons	Lab	Greens	Lib Dem	UKIP	SNP	PC
Total	μ	3.33	5.55	4.36	4.52	1.19	1.80	2.09
	σ	4.02	3.83	3.45	3.37	2.66	3.14	3.22
High Intensity Vignette	μ	3.35	5.67	4.57	4.62	1.25	1.7	2.11
	σ	4.09	3.84	3.48	3.32	2.75	3	3.25
Low Intensity Vignette	μ	3.19	5.41	4.33	4.3	1.06	1.94	2.04
	σ	4.01	3.86	3.5	3.45	2.5	3.32	3.19
Control	μ	3.41	5.54	4.1	4.59	1.25	1.8	2.11
	σ	3.98	3.79	3.37	3.35	2.7	3.2	3.23

Table 5: Descriptive Statistics of the Party Affiliation
