# Supplementary material for: Measuring the effects of differentially intense information on political opinions
Source: PLoS One. 2025 Nov 26;20(11):e0333129. doi: 10.1371/journal.pone.0333129 (PMC12654871; doi:10.1371/journal.pone.0333129)
Supplement: S1 Fig — (PDF) [file pone.0333129.s008.pdf]

1 S1 Fig.: Scree Plot Principal Component Analysis

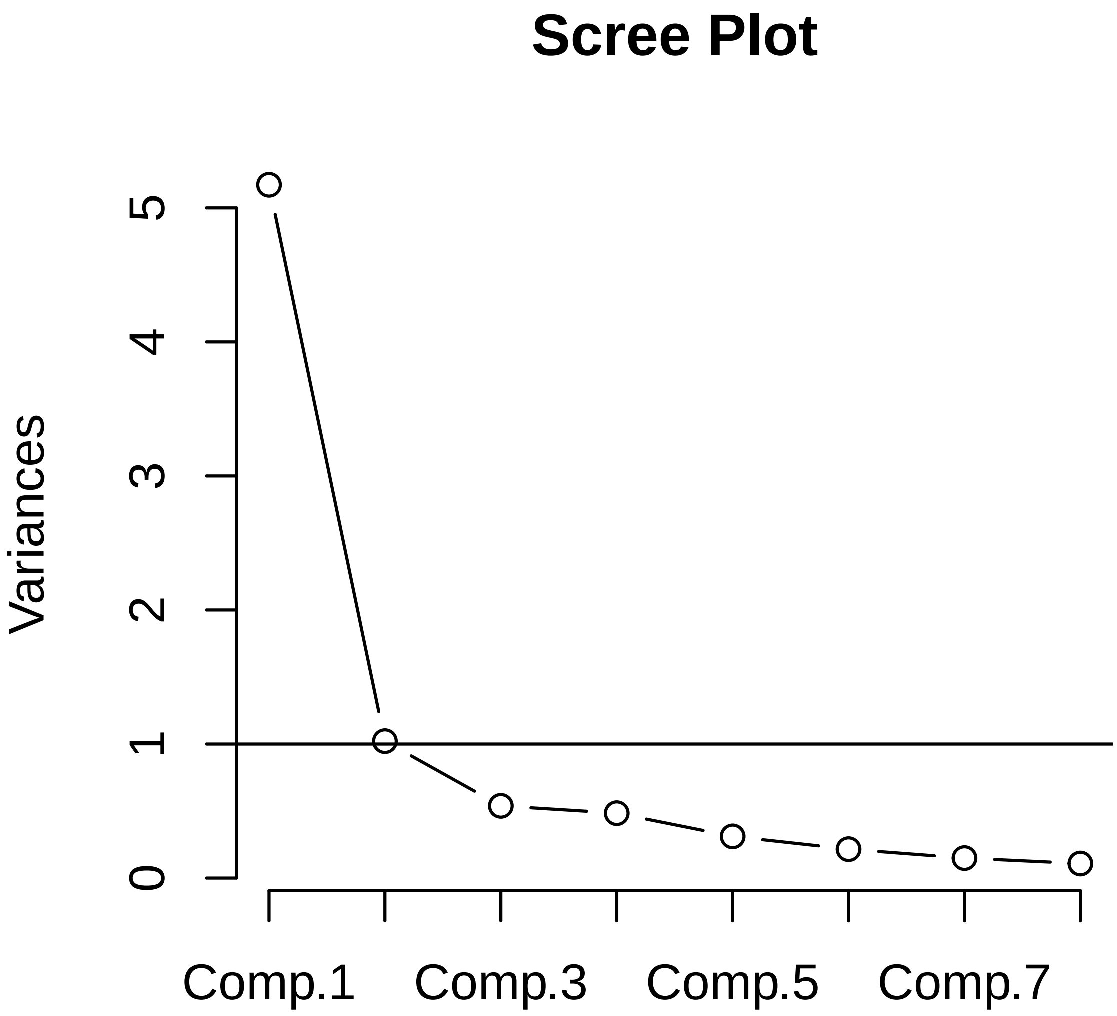

Figure 1: Principal Component Analysis (PCA).

The rotation in the PCA used the Varimax method. Fit based upon of diagonal values = 0.99. The analysis was conducted with the R software environment with the *psych* package, (Revelle, 2018).
