# Supplementary material for: Measuring the effects of differentially intense information on political opinions
Source: PLoS One. 2025 Nov 26;20(11):e0333129. doi: 10.1371/journal.pone.0333129 (PMC12654871; doi:10.1371/journal.pone.0333129)
Supplement: S6 Table — (PDF) [file pone.0333129.s009.pdf]

S6 Table: Average Treatment Effect (ATE) computed with t-test.

Cases	N	$\mu_{Y(1)}$	$\mu_{Y(0)}$	ATE	St. Error
C1 Performance - High Intensity	819	0.035	-0.029	0.064	0.071
C2 Capability - High Intensity	819	0.345	-0.132	0.477***	0.067
C3 Performance - Low Intensity	753	-0.006	-0.029	0.023	0.071
C4 Capability - Low Intensity	753	-0.251	-0.132	-0.119	0.072

Table 6: ATE computed using T test in R base. Table printed using xtable package (Dahl et al., 2019).
