# Supplementary material for: Measuring the effects of differentially intense information on political opinions
Source: PLoS One. 2025 Nov 26;20(11):e0333129. doi: 10.1371/journal.pone.0333129 (PMC12654871; doi:10.1371/journal.pone.0333129)
Supplement: S2 Fig — (PDF) [file pone.0333129.s010.pdf]

2 S2 Fig: Average Treatment Effect (ATE) results visualized

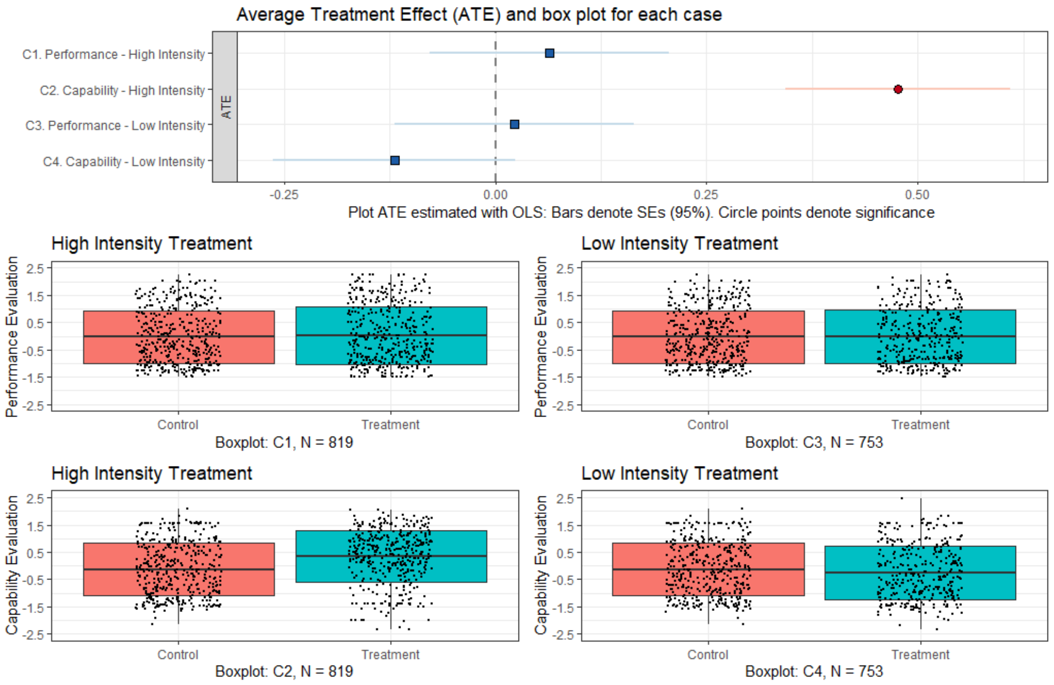

Figure 2: Forest plot displaying ATEs. Boxplots showing the difference between treated and control groups for each of the four cases.
