# Supplementary material for: Measuring the effects of differentially intense information on political opinions
Source: PLoS One. 2025 Nov 26;20(11):e0333129. doi: 10.1371/journal.pone.0333129 (PMC12654871; doi:10.1371/journal.pone.0333129)
Supplement: S3 Fig — (PDF) [file pone.0333129.s011.pdf]

S3 Fig: Average Treatment Effect Analysis with covariates

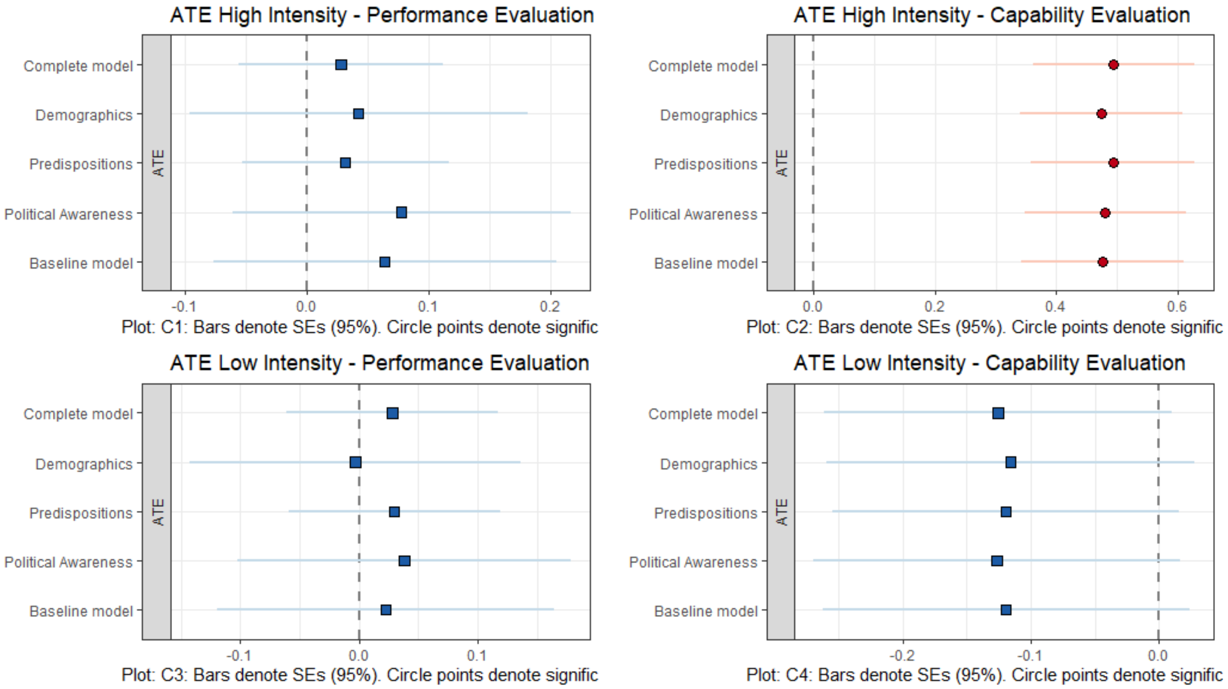

Figure 3: Comparison of Average Treatment Effects including individual level covariates. ATEs are estimated using linear regression (OLS). Bars denote 0.5 (inner) resp. 0.95 (outer) confidence intervals (computed from standard errors). Circle points denote significance. Figure plotted with the R package texreg -GitHub version 1.26.24 (Leifeld, 2013). Covariates are added in blocks: baseline model; political awareness (Interest in Politics, Political Knowledge, Time spent online looking for political information); predispositions (Trust in British politicians, Satisfaction with British Democracy, Party Affiliation); demographics (age, gender, level of education, housing); all of the above together. The difference between the five cases is negligible.
