# Supplementary material for: Measuring the effects of differentially intense information on political opinions
Source: PLoS One. 2025 Nov 26;20(11):e0333129. doi: 10.1371/journal.pone.0333129 (PMC12654871; doi:10.1371/journal.pone.0333129)
Supplement: S7 Table — (PDF) [file pone.0333129.s012.pdf]

## S7 Table: Inbalance Analysis for the treatment with High Intensity

| Sample tested with High Intensity Treatment | $\mu_{Y(0)}$ | $\mu_{Y(1)}$ | Adj. Diff. Null. Sd | Std. Diff | |
|---|---|---|---|---|---|
| Interest in Politics | 0.00 | 0.01 | 0.05 | 0.02 | |
| Knowledge | 0.00 | -0.22 | 0.15 | -0.10 | |
| Time spent online | 0.00 | 0.01 | 0.07 | 0.01 | |
| Satisfaction | 0.00 | 0.12 | 0.09 | 0.09 | |
| Trust | 0.00 | -0.03 | 0.07 | -0.03 | |
| Party Affiliation | 0.00 | -0.12 | 0.18 | -0.05 | |
| Gender | 0.00 | 0.00 | 0.03 | 0.01 | |
| Age | 0.00 | 0.47 | 1.16 | 0.03 | |
| Education | 0.00 | -0.14 | 0.07 | -0.15 | * |
| Housing | 0.00 | -0.08 | 0.09 | -0.06 | |

Table 7: Standardized differences on the unstratified sample computed to check on imbalance between treatment and control groups' variables. The table displays the adjusted means, the standard deviation of the Normal approximated randomization distribution of the strata-adjusted difference of means under the strict null of no effect, standard difference, and pvalues. Estimates computed with the function xBalance from the RItools package (Bowers et al., 2016)
