# Supplementary material for: Measuring the effects of differentially intense information on political opinions
Source: PLoS One. 2025 Nov 26;20(11):e0333129. doi: 10.1371/journal.pone.0333129 (PMC12654871; doi:10.1371/journal.pone.0333129)
Supplement: S8 Table — (PDF) [file pone.0333129.s013.pdf]

S8 Table: Inbalance Analysis for the treatment with Low Intensity

Sample tested with Low Intensity Treatment	$\mu_{Y(0)}$	$\mu_{Y(1)}$	Adj. Diff. Null. Sd	Std. Diff	
Interest in Politics	0.00	0.06	0.05	0.08	
Knowledge	0.00	-0.08	0.15	-0.04	
Time spent online	0.00	0.05	0.07	0.06	
Satisfaction	0.00	-0.00	0.10	-0.00	
Trust	0.00	0.02	0.08	0.02	
Party Affiliation	0.00	0.10	0.20	0.04	
Gender	0.00	0.01	0.04	0.02	
Age	0.00	0.90	1.21	0.05	
Education	0.00	-0.18	0.07	-0.18	*
Housing	0.00	-0.12	0.10	-0.09	
