# Supplementary material for: Measuring the effects of differentially intense information on political opinions
Source: PLoS One. 2025 Nov 26;20(11):e0333129. doi: 10.1371/journal.pone.0333129 (PMC12654871; doi:10.1371/journal.pone.0333129)
Supplement: S4 Fig — (PDF) [file pone.0333129.s014.pdf]

3 S4 Fig.: Difference in control and treatment group in interaction with Knowledge -case3

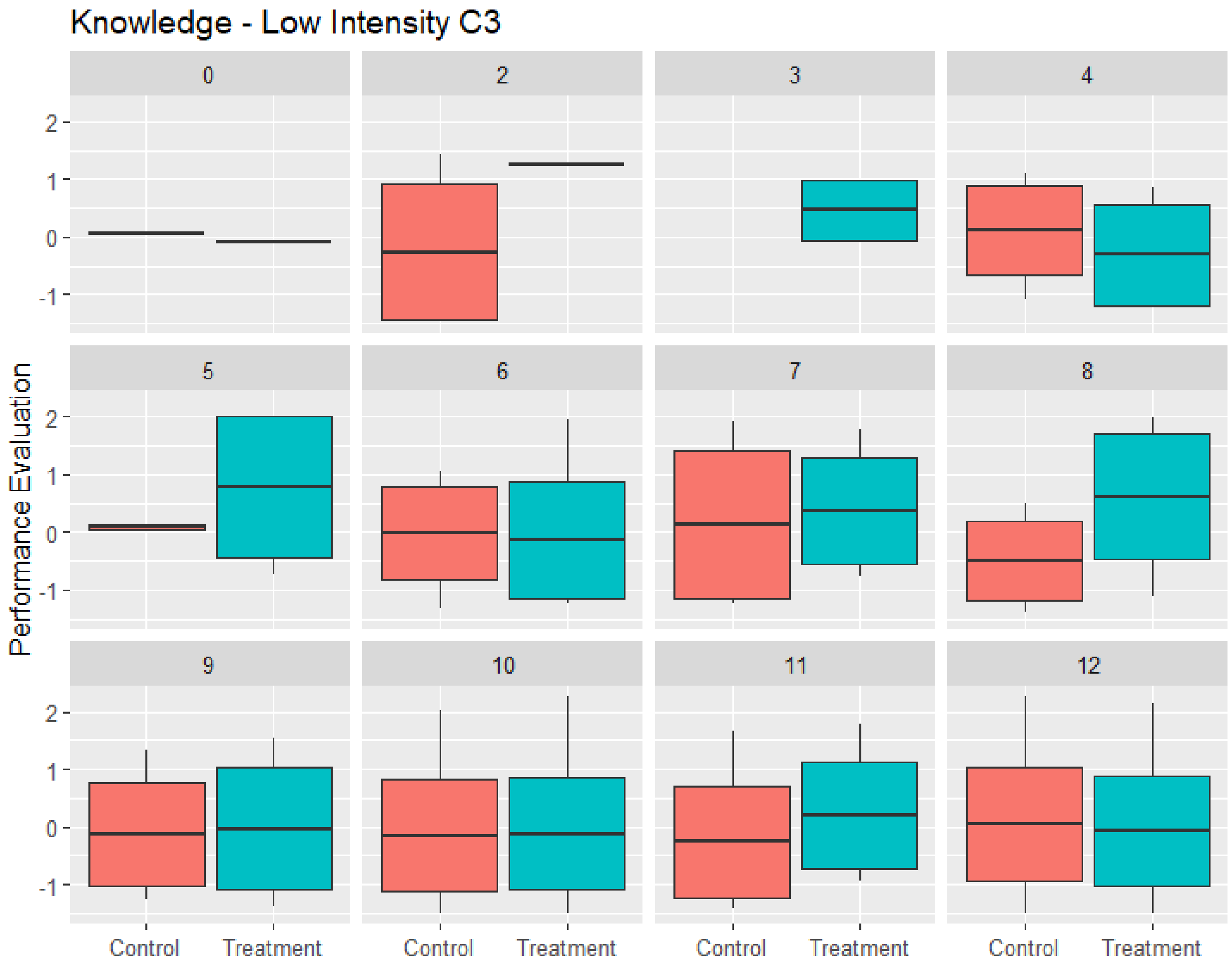

Figure 4: Difference in control and treatment group in interaction with Knowledge -case3
