# Supplementary material for: Measuring the effects of differentially intense information on political opinions
Source: PLoS One. 2025 Nov 26;20(11):e0333129. doi: 10.1371/journal.pone.0333129 (PMC12654871; doi:10.1371/journal.pone.0333129)
Supplement: S5 Fig — (PDF) [file pone.0333129.s015.pdf]

4 S5 Fig.: Difference in control and treatment group in interaction with Satisfaction and Trust -case1

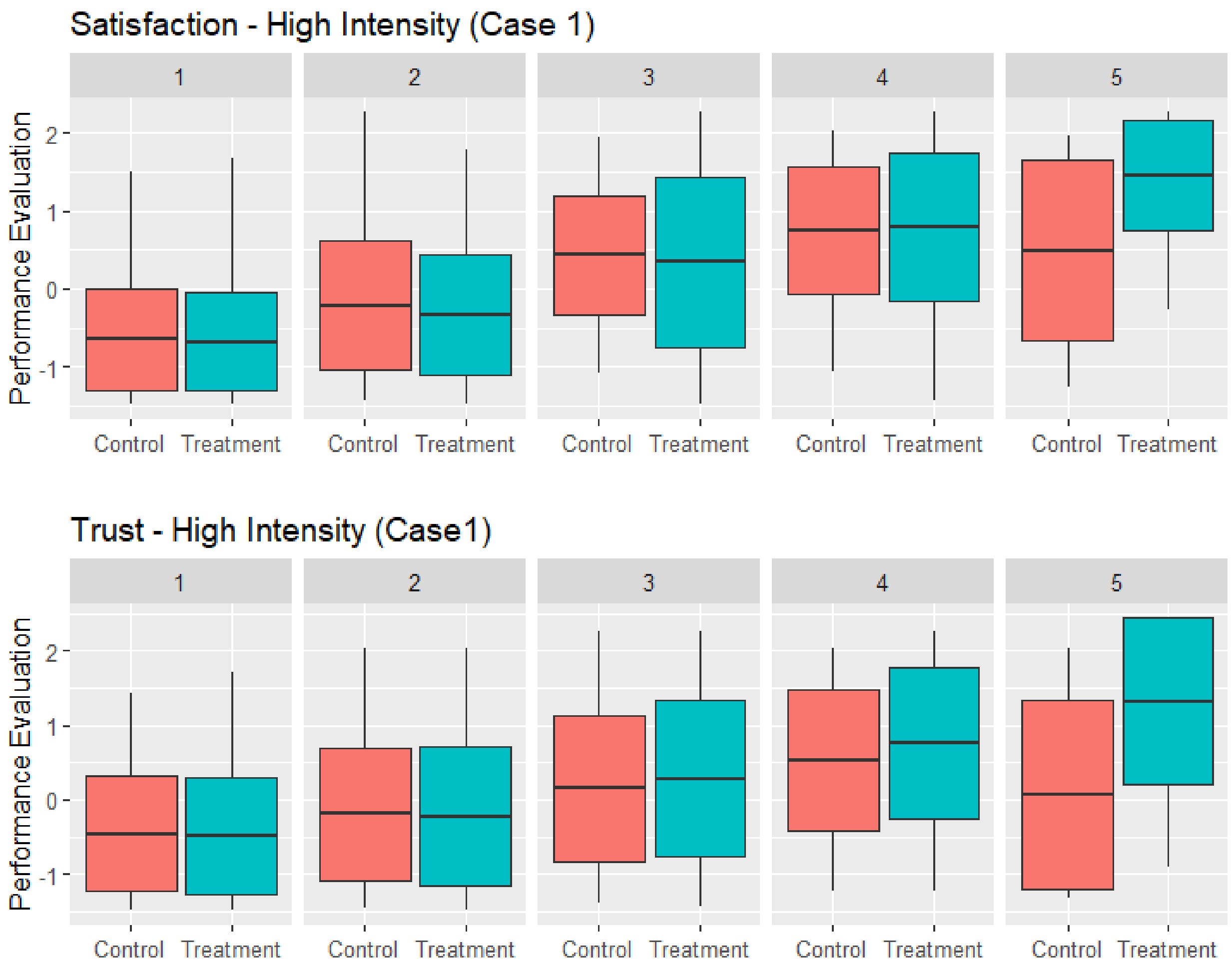

Figure 5: Difference in control and treatment group in interaction with Satisfaction and Trust -case1
