# Supplementary material for: Measuring the effects of differentially intense information on political opinions
Source: PLoS One. 2025 Nov 26;20(11):e0333129. doi: 10.1371/journal.pone.0333129 (PMC12654871; doi:10.1371/journal.pone.0333129)
Supplement: S3 Appendix — (PDF) [file pone.0333129.s016.pdf]

S3 Appendix: Rcode

```
##########################################################################################
#Codepaper:An experimental test of the effects of differentially
# intense information on political opinions
##########################################################################################
# Intensity Estimation
##########################################################################################

high <- c(6, 2, 5, 5, 2, 5, 3, 2)
length(high)

low <- c(2, 2, 1, 2, 1, 2, 1, 1, 1, 2, 2, 3, 2, 2, 2, 1, 1)
length(low)


Phigh <- high / (sum(high))
Plow <- low/ (sum(low))

normalized_entropy <- function(prob_vector) {
  prob_vector <- prob_vector[prob_vector > 0]  # Remove zeros
  H <- -sum(prob_vector * log2(prob_vector))
  H_max <- log2(length(prob_vector))
  H_normalized <- H / H_max
  return(H_normalized)
}

I1 <- 1-normalized_entropy(Phigh)
I2 <- 1-normalized_entropy(Plow)

##########################################################################################
# Simulated Experiment to provide an empirical range to interpret
# the intensity measure
##########################################################################################

### Create a 1000 vectors from size 1-1000 and random frequency.
### Estimate intensity, compare to real observations

# Set seed for reproducibility (optional)
set.seed(123)

# Number of vectors
n_vectors <- 1000

# Initialize list
prob_list <- vector("list", n_vectors)

# Generate vectors
for (i in 1:n_vectors) {
  vec_length <- i  # length from 1 to 200

  # Generate random positive numbers
  random_vals <- runif(vec_length)
```

```
  # Normalize to sum to 1
  prob_vector <- random_vals / sum(random_vals)

  # Store in list
  prob_list[[i]] <- prob_vector
}

# Example: check first few
str(prob_list[1:3])



i <-10

I <- vector("list", 1000)

for (i in 1:length(prob_list)) {

  I[[i]] <- 1-(normalized_entropy(prob_list[[i]]))
  # cat(temp [i], "interation \n")

}

I <- unlist(I)

theme_test <- cbind("N_themes" = 1:1000, "Intensity" = I)

highlight_df <- data.frame(
N_themes = c(length(high), length(low)),
Intensity = c(I1, I2))

q <- quantile(na.omit(theme_test[,2]), probs = c(0.5))

highlight_df$group <- c("high", "low")

ggplot(theme_test, aes(x = N_themes, y = Intensity)) +
  geom_point(alpha = 0.5, size = 1.5) +
  geom_smooth(method = "loess", se = TRUE, color = "blue") +
  geom_hline(yintercept = q, linetype = "dashed", color = "gray50") +
  geom_point(
    data = highlight_df,
    aes(x = N_themes, y = Intensity, color = group, shape = group),
    size = 3
  ) +
  scale_color_manual(
    values = c("high" = "blue", "low" = "red")
  ) +
  scale_shape_manual(
    values = c("high" = 16, "low" = 17)
  ) +
  labs(
    title = "Scatterplot with the estimation of
    intensity for simulated information flows of size 1 to 1000 themes",
    x = "N themes",
```

```r
    y = "Intensity",
    shape = "Point"
  ) +
  guides(color = "none") +
  theme_minimal()

summary(theme_test[,2])




### Simulated experiment 2.
### create 5 sets of 1000 vector each with vector size respectively
### 8, 17, 50, 100, 200 and compare their intensity to the real one
### observed in the Vignettes

# test by sample size (N themes) 8.

# Set seed for reproducibility (optional)
set.seed(123)

# Number of vectors
n_vectors <- 1000
vector_length8 <- 8

# Initialize list
prob_list8 <- vector("list", n_vectors)

# Fill the list
for (i in 1:n_vectors) {
  random_vals8 <- runif(vector_length8)  # random positive numbers
  prob_vector8 <- random_vals8 / sum(random_vals8)   # normalize to sum to 1
  prob_list8[[i]] <- prob_vector8
}

# Example: first vector
print(prob_list8[[1]])
# Check: sums to 1
sum(prob_list8[[1]])

I8 <- vector("list", 1000)

for (i in 1:length(prob_list8)) {

  I8[[i]] <- 1-(normalized_entropy(prob_list8[[i]]))
  # cat(temp [i], "interation \n")

}

I8 <- unlist(I8)


# Example vector: 8 probabilities
set.seed(123)
```

```
#library(ggplot2)

# Example vector
df8 <- data.frame(I8 = I8)


#########################################################################################

# test by sample size (N themes) 17.

# Set seed for reproducibility (optional)
set.seed(123)

# Number of vectors
n_vectors <- 1000
vector_length17 <- 17

# Initialize list
prob_list17 <- vector("list", n_vectors)

# Fill the list
for (i in 1:n_vectors) {
  random_vals17 <- runif(vector_length17)  # random positive numbers
  prob_vector17 <- random_vals17 / sum(random_vals17)  # normalize to sum to 1
  prob_list17[[i]] <- prob_vector17
}

# Example: first vector
print(prob_list17[[1]])
# Check: sums to 1
sum(prob_list17[[1]])

I17 <- vector("list", 1000)

for (i in 1:length(prob_list17)) {

  I17[[i]] <- 1-(normalized_entropy(prob_list17[[i]]))
  # cat(temp[i], "interation \n")

}

I17 <- unlist(I17)


# Example vector: 17 themes and random probabilities
set.seed(123)

#library(ggplot2)

# Example vector
df17 <- data.frame(I17 = I17)
```

```
########################################################################################

# test by sample size (N themes) 50.

# Set seed for reproducibility (optional)
set.seed(123)

# Number of vectors
n_vectors <- 1000
vector_length50 <- 50

# Initialize list
prob_list50 <- vector("list", n_vectors)

# Fill the list
for (i in 1:n_vectors) {
  random_vals50 <- runif(vector_length50)  # random positive numbers
  prob_vector50 <- random_vals50 / sum(random_vals50)  # normalize to sum to 1
  prob_list50[[i]] <- prob_vector50
}

# Example: first vector
print(prob_list50[[1]])
# Check: sums to 1
sum(prob_list50[[1]])

I50 <- vector("list", 1000)

for (i in 1:length(prob_list50)) {

  I50[[i]] <- 1-(normalized_entropy(prob_list50[[i]]))
  # cat(temp [i], "interation \n")

}

I50 <- unlist(I50)


# Example vector: 50 themes and random probabilities
set.seed(123)

#library(ggplot2)

# Example vector
df50 <- data.frame(I50 = I50)



########################################################################################

# test by sample size (N themes) 100.

# Set seed for reproducibility (optional)
```

```r
set.seed(123)

# Number of vectors
n_vectors <- 1000
vector_length100 <- 100

# Initialize list
prob_list100 <- vector("list", n_vectors)

# Fill the list
for (i in 1:n_vectors) {
  random_vals100 <- runif(vector_length100)  # random positive numbers
  prob_vector100 <- random_vals100 / sum(random_vals100)  # normalize to sum to 1
  prob_list100[[i]] <- prob_vector
}

# Example: first vector
print(prob_list100[[1]])
# Check: sums to 1
sum(prob_list100[[1]])

I100 <- vector("list", 1000)

for (i in 1:length(prob_list100)) {

  I100[[i]] <- 1-(normalized_entropy(prob_list100[[i]]))
  # cat(temp[i], "interation \n")

}

I100 <- unlist(I100)


# Example vector: 50 themes and random probabilities
set.seed(123)

#library(ggplot2)

# Example vector
df100 <- data.frame(I100 = I100)



##########################################################################################

# test by sample size (N themes) 200.

# Set seed for reproducibility (optional)
set.seed(123)

# Number of vectors
n_vectors <- 1000
vector_length200 <- 200
```

```r
# Initialize list
prob_list200 <- vector("list", n_vectors)

# Fill the list
for (i in 1:n_vectors) {
  random_vals200 <- runif(vector_length200)  # random positive numbers
  prob_vector200 <- random_vals200 / sum(random_vals200)  # normalize to sum to 1
  prob_list200[[i]] <- prob_vector
}

# Example: first vector
print(prob_list200[[1]])
# Check: sums to 1
sum(prob_list200[[1]])

I200 <- vector("list", 1000)

for (i in 1:length(prob_list200)) {

  I200[[i]] <- 1-(normalized_entropy(prob_list200[[i]]))
  # cat(temp [i], "interation \n")

}

I200 <- unlist(I200)


# Example vector: 50 themes and random probabilities
set.seed(123)

#library(ggplot2)

# Example vector
df200 <- data.frame(I200 = I200)


######################################################################
# unified density plot

# Combine into long data frame
df <- data.frame(
  value = c(I8, I17, I50, I100, I200),
  group = factor(rep(c("8-themes", "17-themes", "50-themes",
  "100-themes", "200-themes"), each = 1000
  )) )

# Special points
x1 <- I1
x2 <- I2

points_df <- data.frame(
  x = c(x1, x2),
  y = c(0, 0),
  type = c("High-Intensity", "Low-Intensity"),
```

```
  color = c("blue", "red")
)

# Plot with separate legends
ggplot() +
  # Density curves with fill and color mapped to group
  geom_density(
    data = df,
    aes(x = value, fill = group, color = group),
    alpha = 0.4
  ) +

  # Special points: map shape to type, but set color manually
  geom_point(
    data = points_df,
    aes(x = x, y = y, shape = type),
    size = 3,
    color = points_df$color
  ) +

  labs(
    title = "Simulation with 1000 iteration each of Information flows with 8, 17, 50, 100, [illegible]
    x = "Intensity",
    y = "Density (how often a certain value of Intensity occurs)",
    fill = "Group",
    color = "Group",
    shape = "Observed Intensity"
  ) +
  theme_minimal()



# Calculate mean of the x-axis
mean_x <- mean(df$value)

# Plot with vertical mean line
ggplot() +
  # Density curves
  geom_density(
    data = df,
    aes(x = value, fill = group, color = group),
    alpha = 0.4
  ) +

  # Special points
  geom_point(
    data = points_df,
    aes(x = x, y = y, shape = type),
    size = 3,
    color = points_df$color
  ) +

  # Vertical line at mean of x-axis
```

```
  geom_vline(
    xintercept = mean_x,
    linetype = "dashed",
    color = "black"
  ) +

  # Optional: add text label for the mean
  annotate(
    "text",
    x = mean_x,
    y = Inf,           # top of plot
    label = "Mean",
    vjust = 2,         # move label down a bit
    hjust = -0.1       # adjust horizontal position if needed
  ) +

  labs(
    title = "Simulation with 1000 iteration each of Information flows
    with 8, 17, 50, 100, and 200 themes and random frequencies",
    x = "Intensity",
    y = "Density (how often a certain value of Intensity occurs)",
    fill = "Group",
    color = "Group",
    shape = "Observed Intensity"
  ) +
  theme_minimal()



length(df$value)

min(df$value) #0.01813037
max(df$value) #0.1651506
mean(df$value) #0.0433017
summary(df$value)
# 1st Qu.0.03198
# median 0.03493
# 3rd Qu 0.04954



##########################################################################
# Experimental data analysis
##########################################################################


library(texreg)
library(xtable)
library(gridExtra)
library(RItools)

exp_est<-read.csv("cleanedDataExperiment.csv",sep = ",",header=TRUE)

####################################################################
```

```
#Data Analysis
##############################################################################


# splitting data by group
groupT1<-exp_est[(exp_est$GroupLabel=="A"),]
groupT2<-exp_est[(exp_est$GroupLabel=="B"),]
groupC<-exp_est[(exp_est$GroupLabel=="C"),]

Analysis1<-rbind(groupT1,groupC)
Analysis2<-rbind(groupT2,groupC)


# Average Treatment Effect ATE and mean in groups with t.test and OLS
Analysis1$GroupLabel<-factor(Analysis1$GroupLabel,levels=c("C","A"),labels=c("Control","Tr
Analysis2$GroupLabel<-factor(Analysis2$GroupLabel,levels=c("C","B"),labels=c("Control","Tr

Analysis1$GroupLabel<-relevel(Analysis1$GroupLabel,ref="Control")
Analysis2$GroupLabel<-relevel(Analysis2$GroupLabel,ref="Control")

testAn1comp1<-t.test(RC1~GroupLabel,data=Analysis1,alternative="two.sided")
OLSAn1comp1<-lm(RC1~GroupLabel,data=Analysis1)

testAn1comp2<-t.test(RC2~GroupLabel,data=Analysis1,alternative="two.sided")
OLSAn1comp2<-lm(RC2~GroupLabel,data=Analysis1)

testAn2comp1<-t.test(RC1~GroupLabel,data=Analysis2,alternative="two.sided")
OLSAn2comp1<-lm(RC1~GroupLabel,data=Analysis2)

testAn2comp2<-t.test(RC2~GroupLabel,data=Analysis2,alternative="two.sided")
OLSAn2comp2<-lm(RC2~GroupLabel,data=Analysis2)

ATEtable<-texreg(OLSAn1comp1,OLSAn1comp2,OLSAn2comp1,OLSAn2comp2)

#####################################
#CATE #
#####################################
Analysis1$PartAffil<-relevel(Analysis1$PartAffil,ref="PTVcons")

#An1comp1

CATEAn1comp1IntPol<-anova(lm(RC1~GroupLabel+Int_pol,data=Analysis1),
lm(RC1~GroupLabel+Int_pol+GroupLabel*Int_pol,data=Analysis1))
CATEAn1comp1Knowledge<-anova(lm(RC1~GroupLabel+Knowledge,data=Analysis1),
lm(RC1~GroupLabel+Knowledge+GroupLabel*Knowledge,data=Analysis1))
CATEAn1comp1time_online<-anova(lm(RC1~GroupLabel+time_online,data=Analysis1),
lm(RC1~GroupLabel+time_online+GroupLabel*time_online,data=Analysis1))
CATEAn1comp1satisf<-anova(lm(RC1~GroupLabel+satisf,data=Analysis1),
lm(RC1~GroupLabel+satisf+GroupLabel*satisf,data=Analysis1))
CATEAn1comp1trust<-anova(lm(RC1~GroupLabel+trust,data=Analysis1),
lm(RC1~GroupLabel+trust+GroupLabel*trust,data=Analysis1))
CATEAn1comp1PartAffil<-anova(lm(RC1~GroupLabel+PartAffil,data=Analysis1),
lm(RC1~GroupLabel+PartAffil+GroupLabel*PartAffil,data=Analysis1))
CATEAn1comp1gender<-anova(lm(RC1~GroupLabel+gender,data=Analysis1),
```

```
lm(RC1~GroupLabel+gender+GroupLabel*gender,data=Analysis1))
CATEAn1comp1age<-anova(lm(RC1~GroupLabel+age,data=Analysis1),
lm(RC1~GroupLabel+age+GroupLabel*age,data=Analysis1))
CATEAn1comp1edu<-anova(lm(RC1~GroupLabel+edu,data=Analysis1),
lm(RC1~GroupLabel+edu+GroupLabel*edu,data=Analysis1))
CATEAn1comp1housing<-anova(lm(RC1~GroupLabel+housing,data=Analysis1),
lm(RC1~GroupLabel+housing+GroupLabel*housing,data=Analysis1))

#An1comp2

CATEAn1comp2IntPol<-anova(lm(RC2~GroupLabel+Int_pol,data=Analysis1),
lm(RC2~GroupLabel+Int_pol+GroupLabel*Int_pol,data=Analysis1))


CATEAn1comp2Knowledge<-anova(lm(RC2~GroupLabel+Knowledge,data=Analysis1),
lm(RC2~GroupLabel+Knowledge+GroupLabel*Knowledge,data=Analysis1))
CATEAn1comp2time_online<-anova(lm(RC2~GroupLabel+time_online,data=Analysis1),
lm(RC2~GroupLabel+time_online+GroupLabel*time_online,data=Analysis1))


CATEAn1comp2satisf<-anova(lm(RC2~GroupLabel+satisf,data=Analysis1),
lm(RC2~GroupLabel+satisf+GroupLabel*satisf,data=Analysis1))


CATEAn1comp2trust<-anova(lm(RC2~GroupLabel+trust,data=Analysis1),
lm(RC2~GroupLabel+trust+GroupLabel*trust,data=Analysis1))

Analysis1$PartAffil<-relevel(Analysis1$PartAffil,ref="PTVcons")
CATEAn1comp2PartAffil<-anova(lm(RC2~GroupLabel+PartAffil,data=Analysis1),
lm(RC2~GroupLabel+PartAffil+GroupLabel*PartAffil,data=Analysis1))

CATEAn1comp2gender<-anova(lm(RC2~GroupLabel+gender,data=Analysis1),
lm(RC2~GroupLabel+gender+GroupLabel*gender,data=Analysis1))

CATEAn1comp2age<-anova(lm(RC2~GroupLabel+age,data=Analysis1),
lm(RC2~GroupLabel+age+GroupLabel*age,data=Analysis1))

CATEAn1comp2edu<-anova(lm(RC2~GroupLabel+edu,data=Analysis1),
lm(RC2~GroupLabel+edu+GroupLabel*edu,data=Analysis1))

CATEAn1comp2housing<-anova(lm(RC2~GroupLabel+housing,data=Analysis1),
lm(RC2~GroupLabel+housing+GroupLabel*housing,data=Analysis1))

#An2comp1#noeffecteveninsubgroups!

CATEAn2comp1IntPol<-anova(lm(RC1~GroupLabel+Int_pol,data=Analysis2),
lm(RC1~GroupLabel+Int_pol+GroupLabel*Int_pol,data=Analysis2))
CATEAn2comp1Knowledge<-anova(lm(RC1~GroupLabel+Knowledge,data=Analysis2),
lm(RC1~GroupLabel+Knowledge+GroupLabel*Knowledge,data=Analysis2))
CATEAn2comp1time_online<-anova(lm(RC1~GroupLabel+time_online,data=Analysis2),
lm(RC1~GroupLabel+time_online+GroupLabel*time_online,data=Analysis2))
CATEAn2comp1satisf<-anova(lm(RC1~GroupLabel+satisf,data=Analysis2),
lm(RC1~GroupLabel+satisf+GroupLabel*satisf,data=Analysis2))
CATEAn2comp1trust<-anova(lm(RC1~GroupLabel+trust,data=Analysis2),
```

```
lm(RC1~GroupLabel+trust+GroupLabel*trust ,data=Analysis2))
CATEAn2comp1PartAffil<-anova(lm(RC1~GroupLabel+PartAffil ,data=Analysis2),
lm(RC1~GroupLabel+PartAffil+GroupLabel*PartAffil ,data=Analysis2))
CATEAn2comp1gender<-anova(lm(RC1~GroupLabel+gender ,data=Analysis2),
lm(RC1~GroupLabel+gender+GroupLabel*gender ,data=Analysis2))
CATEAn2comp1age<-anova(lm(RC1~GroupLabel+age ,data=Analysis2),
lm(RC1~GroupLabel+age+GroupLabel*age ,data=Analysis2))
CATEAn2comp1edu<-anova(lm(RC1~GroupLabel+edu ,data=Analysis2),
lm(RC1~GroupLabel+edu+GroupLabel*edu ,data=Analysis2))
CATEAn2comp1housing<-anova(lm(RC1~GroupLabel+housing ,data=Analysis2),
lm(RC1~GroupLabel+housing+GroupLabel*housing ,data=Analysis2))

#An2comp2#

CATEAn2comp2IntPol<-anova(lm(RC2~GroupLabel+Int_pol ,data=Analysis2),
lm(RC2~GroupLabel+Int_pol+GroupLabel*Int_pol ,data=Analysis2))
CATEAn2comp2Knowledge<-anova(lm(RC2~GroupLabel+Knowledge ,data=Analysis2),
lm(RC2~GroupLabel+Knowledge+GroupLabel*Knowledge ,data=Analysis2))
CATEAn2comp2time_online<-anova(lm(RC2~GroupLabel+time_online ,data=Analysis2),
lm(RC2~GroupLabel+time_online+GroupLabel*time_online ,data=Analysis2))
CATEAn2comp2satisf<-anova(lm(RC2~GroupLabel+satisf ,data=Analysis2),
lm(RC2~GroupLabel+satisf+GroupLabel*satisf ,data=Analysis2))
CATEAn2comp2trust<-anova(lm(RC2~GroupLabel+trust ,data=Analysis2),
lm(RC2~GroupLabel+trust+GroupLabel*trust ,data=Analysis2))
CATEAn2comp2PartAffil<-anova(lm(RC2~GroupLabel+PartAffil ,data=Analysis2),
lm(RC2~GroupLabel+PartAffil+GroupLabel*PartAffil ,data=Analysis2))
CATEAn2comp2gender<-anova(lm(RC2~GroupLabel+gender ,data=Analysis2),
lm(RC2~GroupLabel+gender+GroupLabel*gender ,data=Analysis2))
CATEAn2comp2age<-anova(lm(RC2~GroupLabel+age ,data=Analysis2),
lm(RC2~GroupLabel+age+GroupLabel*age ,data=Analysis2))
CATEAn2comp2edu<-anova(lm(RC2~GroupLabel+edu ,data=Analysis2),
lm(RC2~GroupLabel+edu+GroupLabel*edu ,data=Analysis2))
CATEAn2comp2housing<-anova(lm(RC2~GroupLabel+housing ,data=Analysis2),
lm(RC2~GroupLabel+housing+GroupLabel*housing ,data=Analysis2))


#making table with f stat and pvalue


Interest_in_politics<-c(paste0(round(CATEAn1comp1IntPol$F[2],3),
ifelse(CATEAn1comp1IntPol$`Pr(>F)`[2]<0.001,"***",
ifelse(CATEAn1comp1IntPol$`Pr(>F)`[2]<0.01,"**",
ifelse(CATEAn1comp1IntPol$`Pr(>F)`[2]<0.05,"*",""))))),
paste0(round(CATEAn1comp2IntPol$F[2],3),
ifelse(CATEAn1comp2IntPol$`Pr(>F)`[2]<0.001,"***",
ifelse(CATEAn1comp2IntPol$`Pr(>F)`[2]<0.01,"**",
ifelse(CATEAn1comp2IntPol$`Pr(>F)`[2]<0.05,"*",""))))),
paste0(round(CATEAn2comp1IntPol$F[2],3),
ifelse(CATEAn2comp1IntPol$`Pr(>F)`[2]<0.001,"***",
ifelse(CATEAn2comp1IntPol$`Pr(>F)`[2]<0.01,"**",
ifelse(CATEAn2comp1IntPol$`Pr(>F)`[2]<0.05,"*",""))))),
paste0(round(CATEAn2comp2IntPol$F[2],3),
ifelse(CATEAn2comp2IntPol$`Pr(>F)`[2]<0.001,"***",
ifelse(CATEAn2comp2IntPol$`Pr(>F)`[2]<0.01,"**",
```

```
ifelse(CATEAn2comp2IntPol$`Pr(>F)`[2]<0.05,"*","")))))
)

Knowledge<-c(paste0(round(CATEAn1comp1Knowledge$F[2],3),
ifelse(CATEAn1comp1Knowledge$`Pr(>F)`[2]<0.001,"***",
ifelse(CATEAn1comp1Knowledge$`Pr(>F)`[2]<0.01,"**",
ifelse(CATEAn1comp1Knowledge$`Pr(>F)`[2]<0.05,"*",""))))),
paste0(round(CATEAn1comp2Knowledge$F[2],3),
ifelse(CATEAn1comp2Knowledge$`Pr(>F)`[2]<0.001,"***",
ifelse(CATEAn1comp2Knowledge$`Pr(>F)`[2]<0.01,"**",
ifelse(CATEAn1comp2Knowledge$`Pr(>F)`[2]<0.05,"*",""))))),
paste0(round(CATEAn2comp1Knowledge$F[2],3),
ifelse(CATEAn2comp1Knowledge$`Pr(>F)`[2]<0.001,"***",
ifelse(CATEAn2comp1Knowledge$`Pr(>F)`[2]<0.01,"**",
ifelse(CATEAn2comp1Knowledge$`Pr(>F)`[2]<0.05,"*",""))))),
paste0(round(CATEAn2comp2Knowledge$F[2],3),
ifelse(CATEAn2comp2Knowledge$`Pr(>F)`[2]<0.001,"***",
ifelse(CATEAn2comp2Knowledge$`Pr(>F)`[2]<0.01,"**",
ifelse(CATEAn2comp2Knowledge$`Pr(>F)`[2]<0.05,"*","")))))
)

Time_Online<-c(paste0(round(CATEAn1comp1time_online$F[2],3),
ifelse(CATEAn1comp1time_online$`Pr(>F)`[2]<0.001,"***",
ifelse(CATEAn1comp1time_online$`Pr(>F)`[2]<0.01,"**",
ifelse(CATEAn1comp1time_online$`Pr(>F)`[2]<0.05,"*",""))))),
paste0(round(CATEAn1comp2time_online$F[2],3),
ifelse(CATEAn1comp2time_online$`Pr(>F)`[2]<0.001,"***",
ifelse(CATEAn1comp2time_online$`Pr(>F)`[2]<0.01,"**",
ifelse(CATEAn1comp2time_online$`Pr(>F)`[2]<0.05,"*",""))))),
paste0(round(CATEAn2comp1time_online$F[2],3),
ifelse(CATEAn2comp1time_online$`Pr(>F)`[2]<0.001,"***",
ifelse(CATEAn2comp1time_online$`Pr(>F)`[2]<0.01,"**",
ifelse(CATEAn2comp1time_online$`Pr(>F)`[2]<0.05,"*",""))))),
paste0(round(CATEAn2comp2time_online$F[2],3),
ifelse(CATEAn2comp2time_online$`Pr(>F)`[2]<0.001,"***",
ifelse(CATEAn2comp2time_online$`Pr(>F)`[2]<0.01,"**",
ifelse(CATEAn2comp2time_online$`Pr(>F)`[2]<0.05,"*","")))))
)

Satisfaction<-c(paste0(round(CATEAn1comp1satisf$F[2],3),
ifelse(CATEAn1comp1satisf$`Pr(>F)`[2]<0.001,"***",
ifelse(CATEAn1comp1satisf$`Pr(>F)`[2]<0.01,"**",
ifelse(CATEAn1comp1satisf$`Pr(>F)`[2]<0.05,"*",""))))),
paste0(round(CATEAn1comp2satisf$F[2],3),
ifelse(CATEAn1comp2satisf$`Pr(>F)`[2]<0.001,"***",
ifelse(CATEAn1comp2satisf$`Pr(>F)`[2]<0.01,"**",
ifelse(CATEAn1comp2satisf$`Pr(>F)`[2]<0.05,"*",""))))),
paste0(round(CATEAn2comp1satisf$F[2],3),
ifelse(CATEAn2comp1satisf$`Pr(>F)`[2]<0.001,"***",
ifelse(CATEAn2comp1satisf$`Pr(>F)`[2]<0.01,"**",
ifelse(CATEAn2comp1satisf$`Pr(>F)`[2]<0.05,"*",""))))),
paste0(round(CATEAn2comp2satisf$F[2],3),
ifelse(CATEAn2comp2satisf$`Pr(>F)`[2]<0.001,"***",
ifelse(CATEAn2comp2satisf$`Pr(>F)`[2]<0.01,"**",
```

```
ifelse(CATEAn2comp2satisf$`Pr(>F)`[2]<0.05,"*",""))))
)


Trust<-c(paste0(round(CATEAn1comp1trust$F[2],3),
ifelse(CATEAn1comp1trust$`Pr(>F)`[2]<0.001,"***",
ifelse(CATEAn1comp1trust$`Pr(>F)`[2]<0.01,"**",
ifelse(CATEAn1comp1trust$`Pr(>F)`[2]<0.05,"*",""))))),
paste0(round(CATEAn1comp2trust$F[2],3),
ifelse(CATEAn1comp2trust$`Pr(>F)`[2]<0.001,"***",
ifelse(CATEAn1comp2trust$`Pr(>F)`[2]<0.01,"**",
ifelse(CATEAn1comp2trust$`Pr(>F)`[2]<0.05,"*",""))))),
paste0(round(CATEAn2comp1trust$F[2],3),
ifelse(CATEAn2comp1trust$`Pr(>F)`[2]<0.001,"***",
ifelse(CATEAn2comp1trust$`Pr(>F)`[2]<0.01,"**",
ifelse(CATEAn2comp1trust$`Pr(>F)`[2]<0.05,"*",""))))),
paste0(round(CATEAn2comp2trust$F[2],3),
ifelse(CATEAn2comp2trust$`Pr(>F)`[2]<0.001,"***",
ifelse(CATEAn2comp2trust$`Pr(>F)`[2]<0.01,"**",
ifelse(CATEAn2comp2trust$`Pr(>F)`[2]<0.05,"*",""))))
)

Party_Affiliation<-c(paste0(round(CATEAn1comp1PartAffil$F[2],3),
ifelse(CATEAn1comp1PartAffil$`Pr(>F)`[2]<0.001,"***",
ifelse(CATEAn1comp1PartAffil$`Pr(>F)`[2]<0.01,"**",
ifelse(CATEAn1comp1PartAffil$`Pr(>F)`[2]<0.05,"*",""))))),
paste0(round(CATEAn1comp2PartAffil$F[2],3),
ifelse(CATEAn1comp2PartAffil$`Pr(>F)`[2]<0.001,"***",
ifelse(CATEAn1comp2PartAffil$`Pr(>F)`[2]<0.01,"**",
ifelse(CATEAn1comp2PartAffil$`Pr(>F)`[2]<0.05,"*",""))))),
paste0(round(CATEAn2comp1PartAffil$F[2],3),
ifelse(CATEAn2comp1PartAffil$`Pr(>F)`[2]<0.001,"***",
ifelse(CATEAn2comp1PartAffil$`Pr(>F)`[2]<0.01,"**",
ifelse(CATEAn2comp1PartAffil$`Pr(>F)`[2]<0.05,"*",""))))),
paste0(round(CATEAn2comp2PartAffil$F[2],3),
ifelse(CATEAn2comp2PartAffil$`Pr(>F)`[2]<0.001,"***",
ifelse(CATEAn2comp2PartAffil$`Pr(>F)`[2]<0.01,"**",
ifelse(CATEAn2comp2PartAffil$`Pr(>F)`[2]<0.05,"*",""))))
)

Gender<-c(paste0(round(CATEAn1comp1gender$F[2],3),
ifelse(CATEAn1comp1gender$`Pr(>F)`[2]<0.001,"***",
ifelse(CATEAn1comp1gender$`Pr(>F)`[2]<0.01,"**",
ifelse(CATEAn1comp1gender$`Pr(>F)`[2]<0.05,"*",""))))),
paste0(round(CATEAn1comp2gender$F[2],3),
ifelse(CATEAn1comp2gender$`Pr(>F)`[2]<0.001,"***",
ifelse(CATEAn1comp2gender$`Pr(>F)`[2]<0.01,"**",
ifelse(CATEAn1comp2gender$`Pr(>F)`[2]<0.05,"*",""))))),
paste0(round(CATEAn2comp1gender$F[2],3),
ifelse(CATEAn2comp1gender$`Pr(>F)`[2]<0.001,"***",
ifelse(CATEAn2comp1gender$`Pr(>F)`[2]<0.01,"**",
ifelse(CATEAn2comp1gender$`Pr(>F)`[2]<0.05,"*",""))))),
paste0(round(CATEAn2comp2gender$F[2],3),
ifelse(CATEAn2comp2gender$`Pr(>F)`[2]<0.001,"***",
```

```
ifelse(CATEAn2comp2gender$`Pr(>F)`[2]<0.01,"**",
ifelse(CATEAn2comp2gender$`Pr(>F)`[2]<0.05,"*","")))
)

Age<-c(paste0(round(CATEAn1comp1age$F[2],3),
ifelse(CATEAn1comp1age$`Pr(>F)`[2] < 0.001, "***",
 ifelse(CATEAn1comp1age$`Pr(>F)`[2] < 0.01, "**",
ifelse(CATEAn1comp1age$`Pr(>F)`[2] < 0.05, "*", ""))))),
paste0(round(CATEAn1comp2age$F[2], 3),
ifelse(CATEAn1comp2age$`Pr(>F)`[2] < 0.001, "***",
ifelse(CATEAn1comp2age$`Pr(>F)`[2] < 0.01, "**",
ifelse(CATEAn1comp2age$`Pr(>F)`[2] < 0.05, "*", ""))))),
paste0(round(CATEAn2comp1age$F[2], 3),
ifelse(CATEAn2comp1age$`Pr(>F)`[2] < 0.001, "***",
ifelse(CATEAn2comp1age$`Pr(>F)`[2] < 0.01, "**",
ifelse(CATEAn2comp1age$`Pr(>F)`[2] < 0.05, "*", ""))))),
paste0(round(CATEAn2comp2age$F[2], 3),
ifelse(CATEAn2comp2age$`Pr(>F)`[2] < 0.001, "***",
ifelse(CATEAn2comp2age$`Pr(>F)`[2] < 0.01, "**",
ifelse(CATEAn2comp2age$`Pr(>F)`[2] < 0.05, "*", ""))))
)

Education <- c(paste0(round(CATEAn1comp1edu$F[2], 3),
ifelse(CATEAn1comp1edu$`Pr(>F)`[2] < 0.001, "***",
ifelse(CATEAn1comp1edu$`Pr(>F)`[2] < 0.01, "**",
ifelse(CATEAn1comp1edu$`Pr(>F)`[2] < 0.05, "*", ""))))),
paste0(round(CATEAn1comp2edu$F[2],3),
ifelse(CATEAn1comp2edu$`Pr(>F)`[2]<0.001,"***",
ifelse(CATEAn1comp2edu$`Pr(>F)`[2]<0.01,"**",
ifelse(CATEAn1comp2edu$`Pr(>F)`[2]<0.05,"*",""))))),
paste0(round(CATEAn2comp1edu$F[2],3),
ifelse(CATEAn2comp1edu$`Pr(>F)`[2]<0.001,"***",
ifelse(CATEAn2comp1edu$`Pr(>F)`[2]<0.01,"**",
ifelse(CATEAn2comp1edu$`Pr(>F)`[2]<0.05,"*",""))))),
paste0(round(CATEAn2comp2edu$F[2],3),
ifelse(CATEAn2comp2edu$`Pr(>F)`[2]<0.001,"***",
ifelse(CATEAn2comp2edu$`Pr(>F)`[2]<0.01,"**",
ifelse(CATEAn2comp2edu$`Pr(>F)`[2]<0.05,"*",""))))
)

Housing<-c(paste0(round(CATEAn1comp1housing$F[2],3),
ifelse(CATEAn1comp1housing$`Pr(>F)`[2]<0.001,"***",
ifelse(CATEAn1comp1housing$`Pr(>F)`[2]<0.01,"**",
ifelse(CATEAn1comp1housing$`Pr(>F)`[2]<0.05,"*",""))))),
paste0(round(CATEAn1comp2housing$F[2],3),
ifelse(CATEAn1comp2housing$`Pr(>F)`[2]<0.001,"***",
ifelse(CATEAn1comp2housing$`Pr(>F)`[2]<0.01,"**",
ifelse(CATEAn1comp2housing$`Pr(>F)`[2]<0.05,"*",""))))),
paste0(round(CATEAn2comp1housing$F[2],3),
ifelse(CATEAn2comp1housing$`Pr(>F)`[2]<0.001,"***",
ifelse(CATEAn2comp1housing$`Pr(>F)`[2]<0.01,"**",
ifelse(CATEAn2comp1housing$`Pr(>F)`[2]<0.05,"*",""))))),
paste0(round(CATEAn2comp2housing$F[2],3),
ifelse(CATEAn2comp2housing$`Pr(>F)`[2]<0.001,"***",
```

```
ifelse(CATEAn2comp2housing$`Pr(>F)`[2]<0.01,"**",
ifelse(CATEAn2comp2housing$`Pr(>F)`[2]<0.05,"*",""))))
)

CATEtable<-rbind(Interest_in_politics,Knowledge,Time_Online,Satisfaction,
Trust,Party_Affiliation,Gender,Age,Education,Housing)

colnames(CATEtable)<-c("Overall-HighEfficiency",
"Skill-HighEfficiency","Overall-LowEfficiency","Skill-LowEfficiency")


xtable(CATEtable)
```

References

Bowers, J., Fredrickson, M., and Hansen, B. (2016). *RItools: Randomization Inference Tools*. R package version 0.1-15.

Braun, V. and Clarke, V. (2006). Using thematic analysis in psychology. *Qualitative research in psychology*, 3(2):77–101.

Conservative Party (2016). Building a country that works for everyone - YouTube. https://www.youtube.com/watch?v=NxG3CnX76BQ. (Accessed on 02/27/2018).

Dahl, D. B., Scott, D., Roosen, C., Magnusson, A., and Swinton, J. (2019). *xtable: Export Tables to LaTeX or HTML*. R package version 1.8-4.

Leifeld, P. (2013). texreg: Conversion of statistical model output in r to latex and html tables. *Journal of Statistical Software*, 55(8):1–24.

May, T. (n.d.a). Theresa May –Wikipedia. https://en.wikipedia.org/wiki/Theresa_May. (Accessed on 07/31/2018).

May, T. (n.d.b). Theresa May, member of Parliament for Maidenhead. http://www.tmay.co.uk/. (Accessed on 07/31/2018).

Newsnight BBC (2016). How is Theresa May different to David Cameron? - YouTube. https://www.youtube.com/watch?v=-U4lq6kJ8GM. (Accessed on 02/27/2018).

Revelle, W. (2018). *psych: Procedures for Psychological, Psychometric, and Personality Research*. Northwestern University, Evanston, Illinois. R package version 1.8.12.
